# Two maize Kip-related proteins differentially interact with, inhibit and are phosphorylated by cyclin D–cyclin-dependent kinase complexes

**DOI:** 10.1093/jxb/erx054

**Published:** 2017-03-24

**Authors:** Silvia K. Godínez-Palma, Fernando R. Rosas-Bringas, Omar G. Rosas-Bringas, Elpidio García-Ramírez, Jorge Zamora-Zaragoza, Jorge M. Vázquez-Ramos

**Affiliations:** 1 Facultad de Química, Departamento de Bioquímica, UNAM, Avenida Universidad y Copilco, México DF 04510, México; 2 I. Medizinische Klinik and Poliklinik, Universitätsmedizin der Johannes Gutenberg-Universität Mainz Obere Zahlbacherstr. 63 55131 Mainz, Germany; 3 Department of Plant Sciences, Plant Developmental Biology, Wageningen University, Droevendaalsesteeg 1, Wageningen 6708 PB, The Netherlands

**Keywords:** CDKs, cyclins D, ICK/KRPs, kinase inhibition, KRP phosphorylation, *Zea mays*.

## Abstract

The family of maize Kip-related proteins (KRPs) has been studied and a nomenclature based on the relationship to rice KRP genes is proposed. Expression studies of KRP genes indicate that all are expressed at 24 h of seed germination but expression is differential in the different tissues of maize plantlets. Recombinant KRP1;1 and KRP4;2 proteins, members of different KRP classes, were used to study association to and inhibitory activity on different maize cyclin D (CycD)–cyclin-dependent kinase (CDK) complexes. Kinase activity in CycD2;2–CDK, CycD4;2–CDK, and CycD5;3–CDK complexes was inhibited by both KRPs; however, only KRP1;1 inhibited activity in the CycD6;1–CDK complex, not KRP4;2. Whereas KRP1;1 associated with either CycD2;2 or CycD6;1, and to cyclin-dependent kinase A (CDKA) recombinant proteins, forming ternary complexes, KRP4;2 bound CDKA and CycD2;2 but did not bind CycD6;1, establishing a differential association capacity. All CycD–CDK complexes included here phosphorylated both the retinoblastoma-related (RBR) protein and the two KRPs; interestingly, while KRP4;2 phosphorylated by the CycD2;2–CDK complex increased its inhibitory capacity, when phosphorylated by the CycD6;1–CDK complex the inhibitory capacity was reduced or eliminated. Evidence suggests that the phosphorylated residues in KRP4;2 may be different for every kinase, and this would influence its performance as a cyclin–CDK inhibitor.

## Highlight

Maize Kip-related proteins can be differentially phosphorylated by different cyclin D–cyclin-dependent kinase complexes and this influences their performance as cyclin-dependent kinase inhibitors.

## Introduction

The life cycle in plants is highly coordinated by regulation of cell cycle events and developmental programs. Control of cell cycle progression in eukaryotes is dependent on a conserved molecular machinery consisting of a number of protein kinases known as cyclin-dependent kinases (CDKs) that are activated after binding to regulatory proteins named cyclins (Cyc) (Vidal and Koff, 2000; Vandepoele *et al.*, 2002).

Several additional factors regulate kinase activity in these cyclin–CDK complexes: stimulation of CDK activity after phosphorylation in Thr 161 by a CDK activating kinase (CAK; Murray, 2004); inhibition by phosphorylation of residue Tyr 15 by the WEE1 kinase (Nakagami *et al.*, 2002; Dewitte and Murray, 2003); and inhibition by binding to proteins known as inhibitors of CDKs (ICKs) that arrest cell cycle progression in response to internal or external cues (Verkest *et al.*, 2005).

In mammals, the family of Cip/Kip proteins inhibits a wide range of cyclin–CDK complexes in the G1–S transition (Stals & Inzé, 2001; Leibovitch *et al.*, 2003) and these proteins apparently also function for assembly and stabilization of cyclin–CDK complexes, or even for transporting complexes into the nuclei, processes in which Cip/Kip proteins can also be phosphorylated by the same kinase complexes (McConnell *et al.*, 1999). Thus, cell cycle regulation by these proteins requires physical contact with CDKs (Leibovitch *et al.*, 2003; Lacy *et al.*, 2004) with low concentrations required for complex stabilization or higher concentrations for inhibition of kinase activity (Zhou *et al.*, 2003*a*, *b*).

In plants, two families of related CDK inhibitors have been described. The first is the Arabidopsis plant-specific SIAMESE/SIAMESE RELATED (SIM/SMR) proteins (Churchman *et al.*, 2006; Peres *et al.*, 2007), which are involved in the regulation of endoreduplication events (Kumar *et al.*, 2015), with some members regulating the innate immune response during interaction with pathogens (Hamdoun *et al.*, 2016) and others regulating cell cycle checkpoints in response to DNA damage induced by oxidative stress (reactive oxygen species) (Yi *et al.*, 2014). The second is the inhibitor of CDK (ICK)/Kip-related protein (KRP) family, which shows a limited similarity to mammalian p27^Kip1^ protein (Wang *et al.*, 1997; De Veylder *et al.*, 2001). This is composed of seven subgroups, originally identified in Arabidopsis (Wang *et al.*, 2006, 2007). By sequence alignment and phylogenetic trees, ICK/KRP proteins have been classified into three groups: class A exclusively for dicotyledonous plants, class B only for monocotyledonous plants, and class C present in both mono- and dicotyledonous plants (Torres Acosta *et al.*, 2011).

There are nine motifs that are conserved among ICK/KRP proteins (Torres Acosta *et al.*, 2011). Motifs 1 and 2 are essential for their inhibitory activity since motif 1 is required for CDK binding and kinase inhibition (Zhou *et al.*, 2003*a*), whereas motif 2 is important for interaction with cyclin D (Wang *et al.*, 1998); motif 7 confers nuclear localization to ICK/KRPs (Jakoby *et al.*, 2006; Bird *et al.*, 2007). Little is known about the function of the other motifs.

Overexpression of ICK/KRP genes in plants shows some common phenotypes that include reduction in plant size, serrated leaves, reduction in cell number and cell elongation (De Veylder *et al.*, 2001; Jasinski *et al.*, 2002*b*, 2003; Barrôco *et al.*, 2006; Wang *et al.*, 2007), and in some cases infertility (Zhou *et al.*, 2002).

Different plant tissues express various members of the ICK/KRP family, although with varied levels, suggesting specific regulatory mechanisms (Wang *et al.*, 1998; De Veylder *et al.*, 2001, Jasinski *et al.*, 2002*a*;Ormenese *et al.*, 2004). Also, the differential expression of different ICK/KRPs during the cell cycle suggests phase-specific regulation (Menges *et al.*, 2005), probably indicating a non-redundant role of the different ICK/KRPs during cell proliferation and differentiation (Torres Acosta *et al.*, 2011).

Interaction between ICK/KRPs and other cell cycle proteins has been followed by yeast two-hybrid screening in Arabidopsis (Wang *et al.*, 1997; Lui *et al.*, 2000; Zhou *et al.*, 2002), tobacco (Jasinski *et al.*, 2002*b*, 2003) and *Medicago truncatula* (Pettkó-Szandtner *et al.*, 2006). ICK/KRP proteins inhibit kinase activity of Cyc–CDK complexes in Arabidopsis (Wang *et al.*, 1997; Lui *et al.*, 2000; Verkest *et al.*, 2005), tobacco (Jasinski *et al.*, 2002*a*), maize (Coelho *et al.*, 2005), *Medicago truncatula* (Pettkó-Szandtner *et al.*, 2006) and tomato (Bisbis *et al.*, 2006).

All Arabidopsis ICK/KRPs and at least one from tobacco interact with cyclins D (De Veylder *et al.*, 2001; Jasinski *et al.*, 2002*b*;Zhou *et al.*, 2002) and inhibition of the associated kinase activity reduces cell cycle progression and DNA content, depending on KRP concentration (Verkest *et al.*, 2005). Interaction of KRPs with other Cyc–CDK complexes has also been demonstrated, since in alfalfa ICK/KRPs bind CDKB2;1 (Pettkó-Szandtner *et al.*, 2006) and in maize bind CycA (Coelho *et al.*, 2005).

Little is known about ICK/KRP regulation in plants at the protein level; Zeama;KRP2 and Arath;KRP2 seem to be regulated by proteolysis during endosperm development (*Zea mays*; Coelho *et al.*, 2005) or leaf development (Arabidopsis; Verkest *et al.*, 2005). In Arabidopsis KRP2 is phosphorylated by CDKA1 or CDKB1;1 kinase complexes and thus targeted for proteolysis (Verkest *et al.*, 2005).

DNA endoreduplication is observed in weak overexpressing ICK1/KRP1 or ICK2/KRP2 transgenic Arabidopsis plants resulting in higher ploidy levels (Verkest *et al.*, 2005; Weinl *et al.*, 2005); with high overexpression, cell cycle progression is inhibited and ploidy is strongly reduced as well as cell number. Whereas single Arath ICK/KRP mutants show no evident phenotypes, multiple mutants show diverse morphological changes such as longer cotyledons, leaves, petals and seeds compared with wild type plants. These multiple mutants show more cells but with reduced size in every organ examined (Cheng *et al.*, 2013).

During maize germination, the advance of cell cycle events is required for germination completion and establishment of a plantlet (Vázquez-Ramos and Sánchez, 2003). Our group has demonstrated the presence of different cyclin D–CDK complexes that show differential associated kinase activity during the germination process (Godínez-Palma *et al.*, 2013); moreover, a maize KRP protein (classified as KRP4;2 in this paper) was shown to inhibit kinase activity in CycD-associated CDKs (De Jesús Juárez *et al.*, 2008; Lara-Núñez *et al.*, 2008).

In this work we have studied the expression of maize ICK/KRPs in different tissues and during seed germination and determined the inhibitory activity that two members of this family (KRP1;1 and KRP4;2) have over different CycD–CDK complexes. We have found that both KRPs are phosphorylated by different CycD–CDK complexes and moreover, that KRP4;2, previously phosphorylated by either CycD2;2–CDKA or CycD6;1–CDKA recombinant complexes, exhibits different inhibitory activity on CycD–CDK complexes immunoprecipitated from cells. We discuss the physiological significance of these results.

## Material and methods

### *In silico* analysis

*Arabidopsis thaliana* and *Oryza sativa* ICK/KRP sequences were obtained from GenBank (National Center for Biotechnology Information; https://www.ncbi.nlm.nih.gov/genbank/).

Maize (*Zea mays*) ICK/KRP gene sequences were drawn from Ensembl Plants (http://plants.ensembl.org;Kersey *et al.*, 2016); protein sequences were aligned to Arabidopsis and rice ICK/KRPs query sequences. Four of the maize ICK/KRP genes previously reported corresponded to Zeama;ICK1, Zeama;ICK2 (Coelho *et al.*, 2005); Zeama;ICK3 and Zeama;ICK4 (Torres Acosta *et al*, 2011) and sequences were drawn from GenBank.

For the alignment of maize, rice and Arabidopsis ICK/KRP sequences, Clustal W Multiple Alignment (Thompson and French, 1994) in BioEdit was used; results were kept in FASTA format and were imported to MEGA5.0 (http://en.bio-soft.net/tree/MEGA.html) in order to be analysed with the neighbor-joining method. The phylogenetic tree was built with the FigTree v1.3.1 program (http://tree.bio.ed.ac.uk/software/figtree/). The identity matrix of maize and rice sequences was obtained by using BioEdit and then transformed to percentages.

Identification of motifs and domains was achieved using the PFAM 26.0 site (http://pfam.xfam.org/search), following the methodology proposed by Torres Acosta *et al.*, 2011. The presence of PEST regions was detected with EMBOSS: epestfind (http://emboss.bioinformatics.nl/cgi-bin/emboss/epestfind); putative CDK phosphorylation sites were determined using GPS 2.0 (Group-based Prediction System; Xue *et al.*, 2008) and nuclear localization signals (NLS) by using the cNLS Mapper program (http://nls-mapper.iab.keio.ac.jp/cgi-bin/NLS_Mapper_form.cgi;Kosugi *et al.*, 2009).

### RNA extraction

For ICK/KRP gene expression during maize germination, five imbibition times were chosen (0, 6, 12, 18 and 24 h). Chalqueño maize seeds were used (harvest 2013) and were disinfected with sodium hypochlorite (3%), rinsing with deionized water. Then seeds received a 5% solution of Sin-Bac® (bromo-chloro dimethyl hidantoin). Seeds were placed between paper towels with deionized water and after each imbibition time embryo axes were dissected.

Different plant tissues were used, leaves (base and tips), roots and coleoptile; for this, plantlets were kept in the dark for 3 days at 25 °C and then changed to a photoperiod regime (16 h light/8 h dark) at 25 °C. Plantlet tissues were obtained after 14 d post-germination. Leaf tissue selection was as reported by Li *et al.* (2010), and the second leaf was selected because it complied with the recommended dimensions (~14 cm long). For primary root tissues, the first 2 mm was used and for coleoptile, the tissue surrounding the stem was recovered and used.

The corresponding tissues (100 mg) were frozen in liquid nitrogen; tissues were homogenized and 1 ml of TRIzol Reagent (Invitrogen) was finally added. Extraction was as indicated by the provider, but extracting twice with chloroform. Samples were quantified and monitored by means of agarose gels (1%).

### cDNA preparation

RNA was calibrated loading 400 ng and the concentration was adjusted after densitometric analysis. Contaminant DNA was removed by incubation with 1 U of RQ1 DNase (Promega) per microgram of RNA, 2 µl 10× Reaction Buffer, in a final volume of 20 µl, at 37 °C for 30 min; then, 2 µl of RQ1 DNase Stop Solution (Promega) was added and incubated 10 min at 60 °C to inactivate the enzyme. cDNA was synthesized by adding 1 µg of RNA using the Improm-IITM Reverse Transcription System kit (Promega) following the instructions of the provider.

### PCR amplification

Specific primers were designed for the different genes and the PCR reaction was performed using the JumpStart^TM^ Taq ReadyMix^TM^ enzyme (Sigma-Aldrich). Amplification conditions are shown in Supplementary Table S3 at *JXB* online. cDNA (1 μl) synthesized from total RNA was used as template in a reaction containing 10 μM of each dNTP, 5× Q5 Reaction Buffer and Q5 High-Fidelity DNA polymerase (0.5 U). For cloning of KRPs, the PCR product was monitored in agarose gels (1%); the band corresponding to the cDNA of each ICK/KRP was cut and purified using the GenElute^TM^ Gel Extraction Kit (Sigma-Aldrich), following the provider’s instructions.

### Recombinant proteins

Purified cDNAs corresponding to KRP4;2 and KRP1;1 were ligated to pGEM-T-easy vector (Promega) using the procedure recommended by the manufacturer. Competent *E. coli*, XL1-blue cells were transformed and positive cells were grown in LB medium at 37 °C for 1 h; then cells were centrifuged, the pellet was resuspended (LB–ampicillin) and cells were grown in agar LB–ampicillin supplemented with X-Gal and isopropyl β-D-1-thiogalactopyranoside (IPTG; 0.5 mM).

Selected colonies were grown in LB–ampicillin, cell extracts were prepared and the plasmid was isolated using the Zyppy^TM^ Plasmid Miniprep kit (Biasys). Plasmids cut by *Eco*RI were confirmed by double restriction with *Not*I (NEB) and *Bam*HI (Invitrogen) enzymes and PCR. After double restriction, the resulting fragments were separated by agarose gel electrophoresis (1.2%) and bands were cut and purified. The recovered fragments were ligated to the pPROEX HTb vector (Invitrogen) and these plasmids were transformed into competent *E. coli* XL1-blue cells. Plasmids were recovered and inserts checked again by double restriction and by sequencing. Nucleotide and amino acid sequences were analysed with Translate from ExPASY Bioinformatics Resource Portal (http://expasy.org/tools/) and multiple alignment using Clustal W in BioEdit, respectively.

To purify recombinant His-KRP1;1 and His-KRP4;2 proteins, LB medium plus IPTG was used (100 ml) incubating at 37 °C for 3 h. Bacterial extracts were spun at 2741 *g* for 15 min, and the pellet was resuspended in 5 ml of buffer B (100 mM NaH_2_PO_4_, 10 mM Tris-Cl and 8 M urea, pH 8.0) and incubated for 1 h at room temperature. The lysate was centrifuged at 11 447 *g* for 20 min at room temperature. The supernatant was added to Ni-NTA agarose resin (Qiagen) for 1 h at room temperature with shaking and washed twice with 4 ml buffer C (100 mM NaH_2_PO_4_, 10 mM Tris-Cl and 8 M urea, pH 6.3). Recombinant proteins were eluted with buffer E (100 mM NaH_2_PO_4_, 10 mM Tris-Cl and 8 M urea, pH 4.5).

Renaturalization of recombinant His-KRP1;1 and His-KRP4;2 proteins was by gradual dialysis (6, 4, 2 M urea) in renaturalization buffer (25 mM Tris pH 7.5, 0.25 M NaCl, 0.15% CHAPS, 0.5 mM DTT and 2.5% glycerol) for 6 h in between each dialysis. The last dialysis was performed in kinase buffer (see below) but without ATP.

### Protein extracts from maize embryonic axes

Maize axes were homogenized in a mortar using liquid nitrogen and protein extraction buffer (70 mM Tris–HCl pH 7.5, 1 mM MgCl_2_, 25 mM KCl, 5 mM Na_2_EDTA, 0.25 M sucrose, 7.5 mM DTT, 0.1% Triton X-100, 60 mM β-glycerol phosphate, 50 mM NaF, 200 μM Na_3_VO_4_, 1 mM EGTA and a tablet of Complete Protease Inhibitors; Roche), the homogenate was centrifuged at 16 000 *g* for 1 h at 4 °C and protein concentration was determined by the method of Bradford (1976).

### Production and validation of anti-CycD6;1 antibodies

A purified non-conserved 53 amino acid-long (P239-N291, Accession Number: GRMZM2G050933) CycD6;1 peptide fused to a glutathione *S*-transferase (GST) tag (GST:CycD6-Ag) was administered to male New Zealand White rabbits as follows: day 1, 1.2 mg with incomplete Freund’s adjuvant (IFA), subcutaneously (SC); day 7, 0.5 mg with IFA, SC; day 20, 1.5 mg with IFA, SC; day 27, 4 mg intraperitoneally; day 46, 0.5 mg intravenously. On day 50, the anti-serum was collected and purified by removal of anti-GST antibodies. Purified anti-CycD6;1 antibodies specifically recognized a full length CycD6;1 recombinant protein (see Supplementary Fig. S3). An immunocompetition assay against increasing amounts of the immunogenic antigen fused to GST (GST:CycD6-Ag) was performed and this showed that the only band recognized in maize extracts (33 kDa) corresponded to endogenous CycD6;1.

### Protein immunoprecipitation

Anti-CycD2;2, anti-CycD4;2, anti-CycD5;3, and anti-CycD6;1 antibodies were conjugated with protein-A agarose (Roche, lot. 70439221, dilution 6:20) for 2 h at room temperature. Then, protein extracts from embryo axes imbibed at different times from 0 to 24 h of imbibition were added (300 μg) and the mixture was incubated with shaking for 24 h at 4 °C, centrifuged and washed with buffer A (25 mM Tris–HCl pH 7.5, 125 mM NaCl, 2.5 mM EDTA pH 8.0, 2.5 mM EGTA, 2.5 mM NaF and 0.1% Triton X-100). The resulting proteins were used for kinase activity assays and for kinase activity inhibition assays by maize ICK/KRPs.

### Kinase activity and kinase inhibition assays

The anti-CycD antibody immunoprecipitated protein complex was incubated in kinase buffer (50 mM Tris pH 7.4, 10 mM MgCl_2_, 2 mM EGTA, 2 mM DTT, 25 μM ATP and 5 μCi [γ-^32^P]ATP), at 30 °C for 25 min, using as substrate recombinant Zeama GST-Ct-RBR, His-KRP1;1, His-KRP4;2, or p-His-KRP4;2 proteins. For kinase inhibition assays, recombinant His-KRP1;1, His-KRP4;2, or p-His-KRP4;2 protein was added in increasing concentrations (0.25, 1.25, 2.5, or 5.0 µg, as indicated in figure legends) with pre-incubation for 1.5 h at 4 °C with each kinase complex before adding kinase buffer and substrate. To end the reaction loading buffer–SDS was added and proteins were denatured at 90 °C for 5 min and separated by SDS-PAGE. Purification of GST-Ct-ZmRBR was performed according to Ramírez-Parra *et al.* (1999). Detection was achieved using a Europio cassette (Bio-Rad, Hercules, CA, USA) and the film was scanned using Personal Imager FX equipment (Bio-Rad).

### *In vitro* interaction of recombinant proteins

*E. coli* strains overexpressing the different recombinant proteins obtained in our lab (GST-CDKA, His-KRP4;2, GST-CycD6;1, PeX-CycD6;1 and CycD2;2-MBP), were grown in LB medium with the corresponding antibiotics (see Supplementary Table S4). Cultures (5 ml) were incubated for 16–18 h at 37 °C with shaking and then were added to 100 ml fresh LB medium and incubated at 37 °C with the corresponding antibiotics until OD_600nm_ around 0.5–0.6 was reached. IPTG was then added to induce expression of the recombinant proteins. After induction, growth media were transferred to 250 ml centrifuge bottles, centrifuged at 2741 *g* and the pellet was resuspended in 10 ml of PBS 1× + 0.05% Tween 20. A tablet of Complete Protease Inhibitors was then added and cells were lysed with lysozyme (Sigma-Aldrich), finally adding 6 mM MgCl_2_, 1 mM EDTA and Benzonase (Sigma-Aldrich, 1.5 U ml^–1^), incubating for 30 min at 37 °C.

Lysates were sonicated using a Sonics Vibra Cell apparatus at an amplitude of 25% with five pulses of 20 s per sonication. The lysate was centrifuged at 11 447*g* for 30 min at 4 °C. The supernatant was filtered through 0.45 µm diameter filters; for interaction assays soluble fractions were mixed and incubated with constant shaking for 18 h at 4 °C. Proteins were purified by passing through a column with the appropriate affinity resin for the fused protein (see Supplementary Table S4), using the following buffers: (i) GST, equilibrated: PBS 1× + 0.05% Tween 20; washing: PBS 1× + 0.1% Tween 20; elution: 10 mM reduced glutathione, 50 mM Tris–HCl pH 8.0; (i) maltose binding protein (MBP), equilibrated: buffer 20 mM Tris–HCl (pH 7.4), 0.2 M NaCl, 1 mM EDTA, 0.05% Tween 20; elution: buffer + 10 mM maltose; and (iii) Profinity eXact (PeX) Resin (Bio-Rad), first with phosphate buffer pH 7.2 + 0.1 M NaCl and then 1 ml phosphate buffer pH 7.2 + 0.1 M NaF. Controls for every resin are shown in Supplementary Fig. S5I–III.

### Western blot

Proteins were separated by SDS-PAGE (12%) and transferred to PVDF Immobilon-PSQ transfer membranes (Millipore) by means of a Trans Blot-Electrophoretic Transfer Cell (Bio-Rad) for 1 h. Membranes were blocked with 10% skimmed milk–PBS–Tween 20, for 2 h at 4 °C. Then membranes were incubated with the corresponding antibodies (1:4000 anti-KRP (De Jesús Juárez *et al.*, 2008), 1:1000 anti-KRP1;1, 1:4000 anti-CycD6;1; 1:5000 anti-CDKA;1; 1;35000 anti-MBP, 1;35000 anti-GST, 1:1000 anti-pT-P/pS-P). At the end of the incubation time membranes were washed three times with PBS 1× + Tween 20. Membranes were incubated with the peroxidase-coupled anti-rabbit antibody for 1 h at room temperature and washed three times as before. A chemiluminescent solution was applied and images were detected with a ChemiDoc (Bio-Rad) equipment using the Imagen Lab program. Densitometric analysis was performed using a Fluor-S MultiImager (Bio-Rad) and the statistical analysis with Prism 5.0b software (GraphPad, San Diego CA, USA).

### Phosphorylation of His-KRP4;2

KRP4;2 phosphorylation was carried out by incubating His-KRP4;2 with the previously purified CycD2;2-MBP/His-CDKA or GST-CycD6;1/His-CDKA recombinant complexes; a phosphorylation reaction was performed using cold ATP under the reaction conditions described above. Recombinant complexes were able to phosphorylate KRP4;2 in the absence of a CDK CAK (yeast Cak1, purified according to Harashima and Schnittger, 2012), which when added enhanced CycD–CDK activity, but also phosphorylated CDKA, complicating purification of p-KRP4;2 (see below). Phosphorylation of KRP4;2 was determined by western blot using the anti-pT-P/pS-P antibody (Abcam, cat. no. ab9344, 1:1000 dilution) and also by ^32^P incorporation (Supplementary Fig. S8A, B and Fig. 6).

### Purification of p-His-KRP4;2

After CycD–CDK phosphorylation of KRP4;2, proteins were heat-denatured, loaded to the PhosphoProtein Purification Kit column (Qiagen) and incubated for 30 min at 4 °C in the purification column. Then proteins were eluted, collected, and this procedure repeated five times. In every purification, the amount of resin used was calculated to purify around 25 µg of recombinant p-His-KRP4 protein; column washing conditions were as indicated by the manufacturer, collecting 500 µl fractions. The final elutions were desalted and then were pooled and concentrated by means of Amicon Ultra and protein was quantified. Identification of the phosphorylated protein was achieved by western blot, using the anti-pT-P/pS-P antibody (1:1000 dilution) (see Supplementary Fig. S8A, B). Quantification of p-KRP4;2 protein was by the method of Bradford (1976).

## Results

### *In silico* characterization and nomenclature of maize ICK/KRPs

The search for maize genes (Ensembl plants; http://plants.ensembl.org;Kersey *et al.*, 2016) orthologous to the ICK/KRPs present in rice (*Oryza sativa*) and Arabidopsis revealed the existence of nine genes conserving the carboxyl-terminal region required for inhibition of kinase activity in CDK proteins (Zhou *et al.*, 2003*a*, *b*; accession numbers are shown in Supplementary Tables S1 and S2). Of these, four corresponded to genes previously reported: Zeama;ICK1, Zeama;ICK2 (Coelho *et al.*, 2005), Zeama;ICK3 and Zeama;ICK4 (Torres Acosta *et al*, 2011), and two seem to be duplications of the same gene (now named KRP1;2a and KRP1;2b), since they have identical coding sequence and only differ in the 5′ untranslated region (see Supplementary Fig. S1A), being both in different loci (see Supplementary Table S2).

**Fig. 1.**
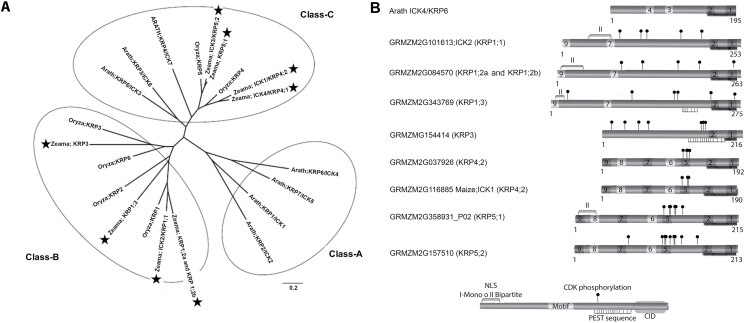
Phylogenetic and sequence analysis of maize ICK/KRP family. (A) Full-length ICK/KRP amino acid sequences from Arabidopsis, *Oriza sativa* and *Zea mays* were aligned and a phylogenetic tree was produced using MEGA4 software. Stars indicate maize ICK/KRP proteins. Their sequences are clustered into two classes (B and C). (B) Schematic overview of putative functional and conserved motifs in maize ICK/KRP proteins. Black circles, phosphorylation motifs; upper bars, mono- or bipartite nuclear localization signal (NLS) motifs; lower bars, PEST boxes. Sequences are organized according to the phylogenetic relationship.

Plant ICK/KRPs (including maize genes) have been classified according to their chronological appearance; however, in this work we propose a nomenclature for maize genes that is based on percentage of identity and phylogenetic comparison particularly with rice genes (Fig. 1A and Supplementary Fig. S1B). Thus, four sequences seem to be more related to Orysa;KRP1, one to Orysa;KRP3, two to Orysa;KRP4 and two to Orysa;KRP5; there were no sequences related to Orysa;KRP2 and Orysa;KRP6 (Fig. 1A and Supplementary Fig. S1B).

The sequences of the nine ICK/KRP proteins were aligned to those corresponding to rice and Arabidopsis genes and a phylogenetic tree was constructed. Figure 1A shows that maize proteins are grouped in two classes previously defined: ICK2/KRP1;1, KRP1;2a, KRP1;2b, KRP1;3 and KRP3 in class B (only monocotyledonous plants), and ICK4/KRP4;1, ICK1/KRP4;2, ICK3/KRP5;1 and KRP5;2 in class C (mono- and dicotyledonous plants).

Protein sequence analysis allowed us to determine the presence of six of the nine motifs already reported for other ICK/KRP proteins (Torres Acosta *et al.*, 2011; Supplementary Fig. S2A) including motifs 1 and 2, located in the carboxyl terminus, required for CDK and cyclin D binding (CDK–cyclin interacting/inhibiting domain; Fig. 1B).

Motifs 3 and 4 were not found in maize KRPs, since these appear to be exclusive to class A proteins (dicotyledonous). On the other hand, proteins with motifs 5, 6 and 8 are grouped in class C in maize, whereas these motifs in rice proteins are distributed in classes B and C (Torres Acosta *et al.*, 2011). Motif 7 is present in proteins of classes B and C, except KRP3, which only contains motifs 1 and 2 (Fig. 1B).

This analysis shows that half of the maize protein sequences have a nuclear localization sequence (NLS) mono- or bipartite, only two maize ICK/KRPs have putative PEST sequences and all proteins present several CDK phosphorylation sites (see Supplementary Fig. S2B).

### Expression of maize ICK/KRP genes during seed germination and in tissues

Expression of maize ICK/KRP genes was monitored from 0 to 24 h of germination and in different plantlet tissues. Specific primers were designed for each ICK/KRP (RT-PCR, Supplementary Table S3); the level of expression was normalized by using expression of the 18S rDNA gene as control. Since KRP1;2a and KRP1;2b genes are identical, primers amplify both transcripts.

**Fig. 2.**
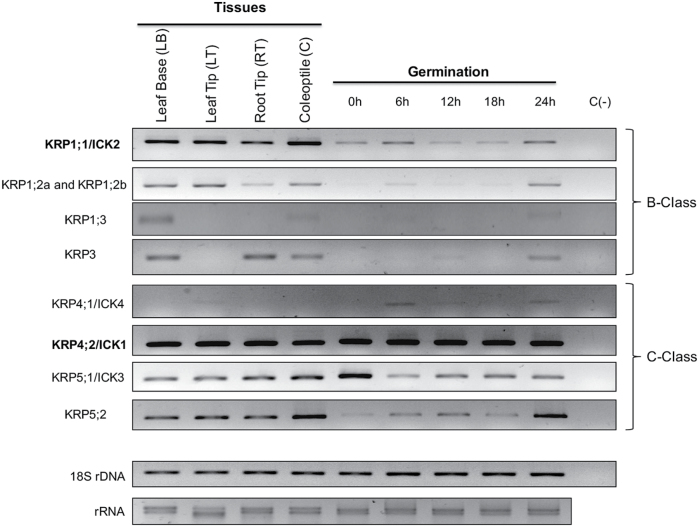
Expression (RT-PCR) of maize ICK/KRP genes during germination, and in different plantlet tissues after 14 days post-imbibition. Germination: 0, 6, 12, 18 and 24 h of imbibition. Genes are organized according to phylogenetic relationship. Class B genes: monocotyledonous seeds; class C genes: monocotyledonous and dicotyledonous seeds. Representative image of three independent biological replicates.

Transcripts of all ICK/KRPs were detected in at least one plantlet tissue (Fig. 2), and expression patterns seem to vary for all ICK/KRPs. KRP1;1, KRP1;2a, KRP1;2b, KRP4;2, KRP5;1, and KRP5;2 were expressed in the four tissues analysed, whereas KRP1;3 and KRP3 were only expressed in leaf base (proliferating zone) and KRP4;1 was only expressed, at low levels, in the leaf tip (differentiation zone) (Sekhon *et al.*, 2011).

During germination all ICK/KRP genes were expressed at 24 h; expression of KRP1;1 and KRP4;2 seemed not to vary along germination but there was a variable expression for all other genes. KRP1;2, KRP1;3, KRP3, and KRP4;1 were not detected in dry embryos. There was no correlation between the pattern of expression and the phylogenetic class to which every ICK/KRP belongs (Fig. 2).

### Inhibition of kinase activity in CycD–CDK complexes by KRP1;1 and KRP4;2 proteins

Previously, we reported differential kinase activity in several CycD–CDK complexes during maize germination (Godínez-Palma *et al.*, 2013), and suggested inhibition of kinase activity by KRPs as a possible mechanism to explain the results obtained. In this paper, two representative members of different phylogenetic classes (KRP1;1, class B and KRP4;2, class C), which are expressed at all germination times, were studied following their inhibitory activity on kinase activity in CycD–CDK complexes.

Specific anti-maize CycD antibodies were used to immunoprecipitate CycsD (CycD2;2, Gutiérrez *et al.*, 2005; CycD4;2, CycD5;3, Godínez-Palma *et al.*, 2013; CycD6;1, Supplementary Fig. S3) from extracts of embryo axes imbibed for different times (0, 6, 12, 18 and 24 h), and then the complexes obtained, composed of Cyc D2;2, D4;2, D5;3 or D6;1 and a CDK, were incubated with increasing concentrations of recombinant Zeama His-KRP1;1 or Zeama His-KRP4;2 proteins (0.25, 2.5, 5.0 µg) to measure kinase activity. Zeama GST-Ct-RBR protein was used as phosphorylation substrate (kinase activity controls using pRBR as target are shown in Supplementary Fig. S4).

**Fig. 3.**
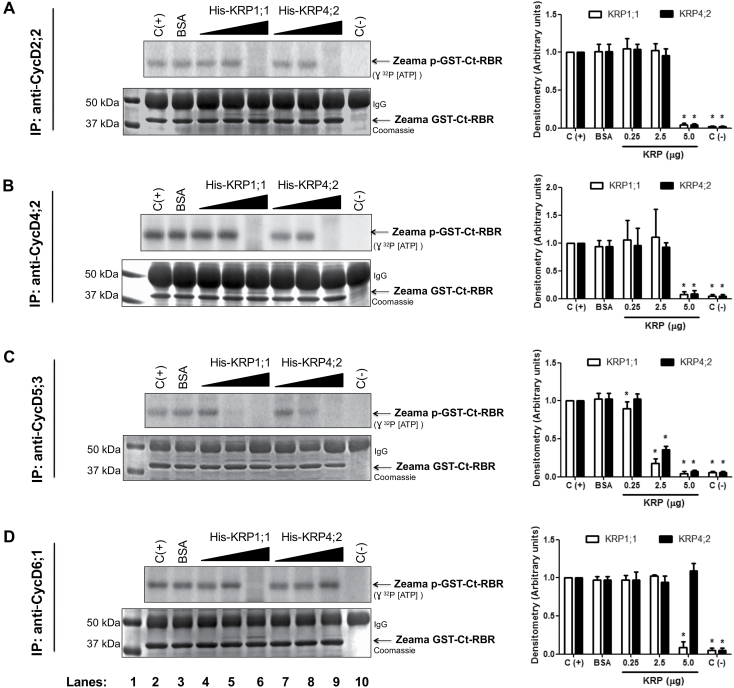
Inhibition of kinase activity in CycD–CDK complexes by KRP1;1 and KRP4;2. Effect of KRP1;1 and KRP4;2 on kinase activity in (A) CycD2;2–CDK complexes, (B) CycD4;2–CDK complexes, (C) CycD5;3–CDK complexes, and (D) CycD6;1–CDK complexes. Lane 1: protein molecular mass markers. Lane 2: immunoprecipitate (IP) using the corresponding anti-CycD antibody without recombinant KRP. Lane 3: IP using the corresponding anti-CycD antibody incubated with 5.0 µg BSA. Lanes 4–6: IPs using the corresponding anti-CycD antibodies incubated with 0.25, 2.5 or 5.0 µg recombinant KRP1;1, respectively. Lanes 7–9: IPs using anti-CycD antibodies incubated with 0.25, 2.5 or 5.0 µg recombinant KRP4;2, respectively. Lane 10: IP using anti-CycD antibodies without GST-Ct-RBR or KRP added. Coomassie Blue stained gels were used as loading control. Densitometry analysis was performed relating band intensity of all samples to the intensity of the loading control and then to the positive control band. Each bar represents the mean±SE from three independent biological replicates. *Statistically significant value (*P*<0.001) compared with control.

Kinase activity in CycD–CDK complexes was reduced by KRP1;1 compared with controls receiving BSA (5.0 µg) or not receiving the KRP protein. Activity in complexes formed by CycD2;2–CDK, D4;2–CDK and D6;1–CDK (Fig. 3A, B, D) disappeared when these were incubated with the highest amount of recombinant KRP (5.0 μg), whereas activity in complexes with CycD5;3 was reduced with 2.5 μg and was no longer detectable at the highest concentration (Fig. 3C).

On the other hand using KRP4;2, inhibition of kinase activity was observed in CycD2;2–CDK, D4;2–CDK and D5;3–CDK complexes, again at the highest concentration; however, CycD6;1–CDK activity was not reduced at any concentration (Fig. 3D, lane 9).

### Complex formation between CycsD, CDKA and KRPs

In mammals, the Cip/Kip family of kinase inhibitors associates to Cycs and CDKs forming different heterotrimeric complexes that differ during cell cycle progression and constitute a critical mechanism to control a successful cell proliferation (Plavletich, 1999).

The differential inhibition of kinase activity observed for KRP4;2 suggested that this KRP interacts differentially with CycD–CDK complexes. For this reason, interaction between cyclins D, CDKA and KRPs was studied. *In vitro* protein–protein interactions were performed by using recombinant proteins bound to distinct protein tags (CycD2;2-MBP, PeX-CycD6;1, GST-CycD6;1, GST-CDKA, His-KRP1;1, and His-KRP4;2). Recombinant proteins were independently induced with IPTG, and the corresponding soluble fractions were mixed and incubated for 18 h. Then different affinity columns allowed us to retain the specific fusion protein and in this way to identify, using specific antibodies, the corresponding coeluting protein(s). Experimental conditions and controls are shown in Supplementary Table S4 and Supplementary Fig. S5.

**Fig. 4.**
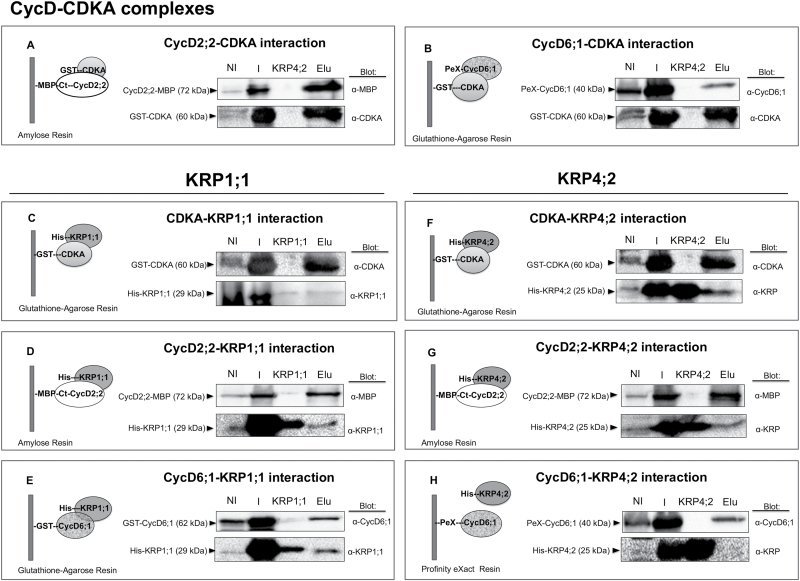
*In vitro* interaction assays between CycD2;2, CycD6;1, CDKA, KRP1;1 and KRP4;2 using different resins to purify and coelute recombinant protein complexes. (A) CycD2;2-MBP–GST-CDKA interaction; CycD2;2-MBP as resin-bound protein. (B) PeX-CycD6;1–GST-CDKA interaction; GST-CDKA as resin-bound protein. (C) GST-CDKA–His-KRP1;1 interaction; GST-CDKA as resin-bound protein. (D) CycD2;1-MBP–His-KRP1;1 interaction; CycD2;1-MBP as resin-bound protein. (E) GST-CycD6;1–His-KRP1;1 interaction; GST-CycD6;1 as resin-bound protein. (F) GST-CDKA–His-KRP4;2 interaction; GST-CDKA as resin-bound protein. (G) CycD2;2-MBP–His-KRP4;2 interaction; CycD2;2-MBP as resin-bound protein. (H) No interaction between PeX-CycD6;1 and His-KRP4;2; PeX-CycD6;1 as resin-bound protein. The following resins were used for complex purification: GST: glutathione agarose resin; MBP: amylose resin; PeX: Profinity eXact Resin. Western blots were performed using anti-CDKA, anti-MBP, anti-KRP1;1, anti-KRP or anti-CycD6;1 antibodies. NI: not induced (no IPTG added); I: induced (IPTG added for protein induction); KRP1;1: purified His-KRP1;1 (added as positive control for KRP identification); KRP4;2: purified His-KRP4;2 (added as positive control for KRP identification); Elu: elution of resin-bound proteins. Representative image of three independent biological replicates.

Dimers formed by proteins GST-CDKA–CycD2;2-MBP (Fig. 4A), GST-CDKA–His-KRP1;1 (Fig. 4C), CycD2;2-MBP–His-KRP1;1 (Fig. 4D), GST-CDKA–His-KRP4;2 (Fig. 4F) and CycD2;2-MBP–His-KRP4;2 (Fig. 4G) could be detected (complete figures are shown in Supplementary Fig. S6); ternary complexes could also be formed consisting of CycD2;2-MBP–GST-CDKA–His-KRP1;1 (see Supplementary Fig. S7A) and CycD2;2-MBP–GST-CDKA–His-KRP4;2 (Supplementary Fig. S7B), since interaction assays indicated that these proteins coeluted.

On the other hand, CycD6;1 bound His-KRP1;1 (Fig. 4E) and GST-CDKA (Fig. 4B), but did not bind His-KRP4;2 (Fig. 4H; complete figures are shown in Supplementary Fig. S6). However, coelution experiments indicated the presence of the trimers PeX-CycD6;1–GST-CDKA–His-KRP1;1 (see Supplementary Fig. S7C) and PeX-CycD6;1–GST-CDKA–His-KRP4;2 (Fig. S7D, E). This suggested that CycD6;1 binds both GST-CDKA and His-KRP1;1, whereas for His-KRP4;2, the trimeric interaction is mediated by GST-CDKA.

### Phosphorylation of KRP1;1 and KRP4;2 by CycD–CDK complexes

**Fig. 5.**
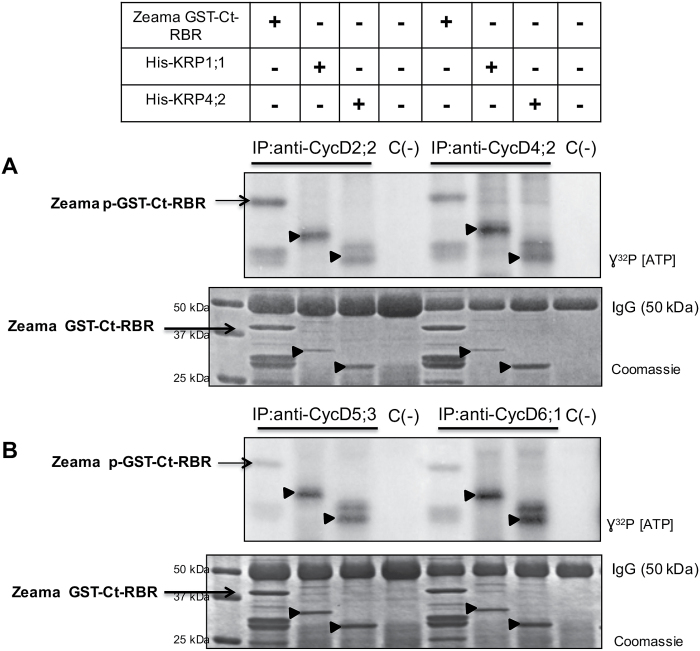
KRP1;1 and KRP4;2 phosphorylation by CycD–CDK complexes. (A) Phosphorylation of Zeama GST-Ct-RBR, His-KRP1;1 and His-KRP4;2 by CycD2;2–CDK and CycD4;2–CDK complexes. (B) Phosphorylation of Zeama GST-Ct-RBR, His-KRP1;1 and His-KRP4;2 by CycD5;3–CDK and CycD6;1–CDK complexes. C(–): anti-CycD immunoprecipitates without Zeama GST-Ct-RBR or KRP added. Arrowheads indicate phosphorylated KRP proteins and recombinant proteins in Coomassie gels that were used as loading control. Representative image of three independent biological replicates.

Putative CDK phosphorylation sites in maize ICK/KRP sequences suggested that these proteins may be targets of the different CycD–CDK complexes. Thus, kinase activity assays were performed using anti-CycD immunoprecipitates from embryo axes as the kinase source and recombinant His-KRP1;1 and His-KRP4;2 as targets. Anti-Cycs D2;2, D4;2, D5;3 and D6;1 immunoprecipitates phosphorylated not only Zeama GST-Ct-RBR but also KRP1;1 and KRP4;2 proteins (Fig. 5A, B).

### Recombinant CycD2;2/CDKA and CycD6;1/CDKA complexes differentially phosphorylate recombinant KRP4;2

**Fig. 6.**
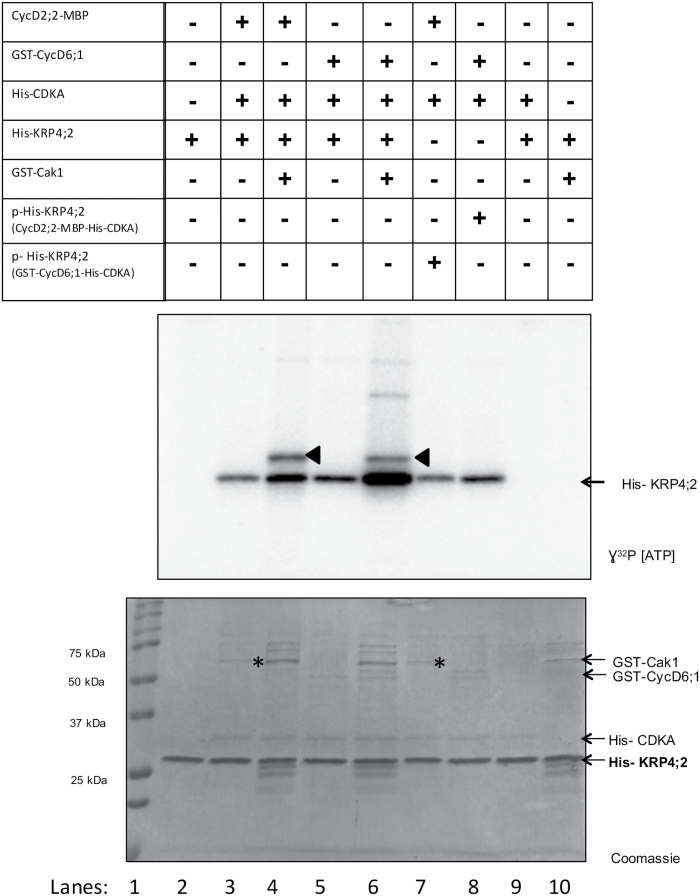
His-KRP4;2 phosphorylation by recombinant CycD2;2-MBP–His-CDKA and GST-Cyc-D6;1–His-CDKA complexes. Lane 1: protein molecular mass markers. Lane 2: His-KRP4;2 in kinase buffer and radioactive ^32^P in the absence of a CycD–CDKA complex. Lane 3: His-KRP4;2 phosphorylation by CycD2;2-MBP–His-CDKA complex. Lane 4: His-KRP4;2 phosphorylation by CycD2;2-MBP–His-CDKA–GST-Cak1 complex. Lane 5: His-KRP4;2 phosphorylation by GST-CycD6;1–His-CDKA complex. Lane 6: His-KRP4;2 phosphorylation by GST-CycD6;1–His-CDKA–GST-Cak1 complex. Lane 7: p-His-KRP4;2 (phosphorylated by GST-CycD6;1–His-CDKA, cold ATP) phosphorylation by CycD2;2-MBP–His-CDKA complex. Lane 8: p-His-KRP4;2 (phosphorylated by CycD2;2-MBP–His-CDKA, cold ATP) phosphorylation by GST-CycD6;1–His-CDKA complex. Lane 9: His-KRP4;2 in kinase buffer and His-CDKA. Lane 10: His-KRP4;2 in kinase buffer and GST-Cak1. The Coomassie Blue stained gel was used as loading control. The band corresponding to CycD2;2-MBP is indicated with an asterisk. Representative image of three independent biological replicates.

Reports indicate that Arabidopsis CycD–CDK complexes *in vitro* phosphorylate histone H1 and phosphorylation is higher if complexes are preincubated with *S. cerevisiae* Cak1 kinase (Harashima and Schnittger, 2012). Figure 6 shows that maize CycD2;2–CDKA and CycD6;1–CDKA recombinant complexes, by themselves, phosphorylated KRP4;2 (lanes 3 and 5); addition of Cak1 kinase caused CDKA phosphorylation (arrowheads, lanes 4 and 6) and also an increase in KRP4;2 phosphorylation, probably due to CDKA activity stimulation. Neither CDKA nor Cak1 alone phosphorylated KRP4;2 (Fig. 6, lanes 9 and 10). This result raised the question of the phosphorylation status of KRP4;2 and its inhibitory capacity.

CycD2;2-MBP/His-CDKA or GST-CycD6;1/His-CDKA recombinant complexes were used to *in vitro* phosphorylate His-KRP4;2 using cold ATP. His-KRP4;2 was then purified using a phospho-affinity resin; Cak1 was not added in this kinase assay to avoid the presence of a phosphorylated CDK. Phosphorylation was demonstrated by western blot using an anti-pT-P/pS-P antibody (see Supplementary Fig. S8A, B).

Purified p-His-KRP4;2 (cold ATP) was used as substrate in kinase assays using different combinations. When GST-CycD6;1–His-CDKA-phosphorylated His-KRP4;2 (p-His-KRP4;2^(GST-CycD6;1/His-CDKA)^) was added to a CycD2;2-MBP–His-CDKA complex, p-His-KRP4;2 was re-phosphorylated as shown by ^32^P labeling (Fig. 6; lane 7). CycD2;2-MBP–His-CDKA-phosphorylated His-KRP4;2 (p-His-KRP4;2^(CycD2;2-MBP/His-CDKA)^) was also re-phosphorylated by the GST-CycD6;1–His-CDKA complex (^32^P labeling, Fig. 6, lane 8). However, re-incubating p-His-KRP4;2 with the initially phosphorylating complex did not produce ^32^P incorporation (Supplementary Fig. S9, lanes 5 and 8). This result indicates that CycD2;2-MBP–His-CDKA and GST-CycD6;1–His-CDKA complexes phosphorylate His-KRP4;2 at more than one residue.

### Differential KRP4;2 phosphorylation by CycD–CDK complexes modifies its inhibitory capacity

To evaluate the inhibitory capacity of phosphorylated His-KRP4;2 by either of the kinase complexes, p-His-KRP4;2 was added to kinase assays using anti-CycD2;2–CDK or anti-CycD6;1–CDK immunoprecipitates from embryo axes.

**Fig. 7.**
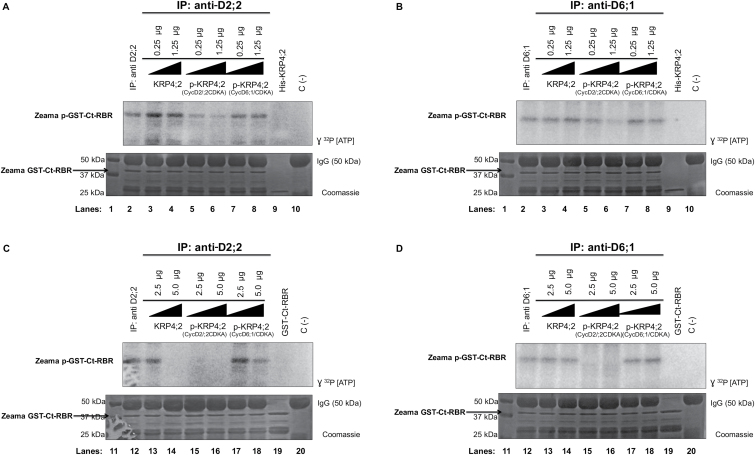
Inhibitory activity of p-His-KRP4;2 on kinase activity in CycD–CDK complexes. (A) Effect of His-KRP4;2 and p-His-KRP4;2 (0.25 and 1.25 µg) on kinase activity in CycD2;2–CDK complexes. (B) Effect of His-KRP4;2 and p-His-KRP4;2 (0.25 and 1.25 µg) on kinase activity in CycD6;1–CDK complexes. (C) Effect of His-KRP4;2 and p-His-KRP4;2 (2.5 and 5.0 µg) on kinase activity in CycD2;2–CDK complexes. (D) Effect of His-KRP4;2 and p-His-KRP4;2 (2.5 and 5.0 µg) on kinase activity in CycD6;1–CDK complexes. Lanes 1 and 11: protein molecular mass markers. Lanes 2 and 12: immunoprecipitates (IP) using anti-CycD antibodies, no recombinant KRP added. Lanes 3 and 4: anti-CycD IPs incubated with 0.25 and 1.25 µg recombinant His-KRP4;2. Lanes 5 and 6: anti-CycD IPs incubated with 0.25 and 1.25 µg recombinant p-His-KRP4;2 (phosphorylated by recombinant CycD2;2-MBP–His-CDKA). Lanes 7 and 8: anti-CycD IPs incubated with 0.25 and 1.25 µg of recombinant p-His-KRP4;2 (phosphorylated by recombinant GST-CycD6;1–His-CDKA). Lane 9: His-KRP4;2 protein incubated with reaction mixture. Lanes 10 and 20: negative control, anti-CycD IP without KRP or RBR substrates. Lanes 13 and 14: anti-CycD IPs incubated with 2.5 and 5.0 µg of recombinant His-KRP4;2 protein. Lanes 15 and 16: anti-CycD IPs incubated with 2.5 and 5.0 µg of recombinant p-His-KRP4;2 protein (phosphorylated by recombinant CycD2;2-MBP–His-CDKA). Lanes 17 and 18: anti-CycD IPs incubated with 2.5 and 5.0 µg of recombinant p-His-KRP4;2 protein (phosphorylated by recombinant GST-CycD6;1–His-CDKA). Lane 19: Zeama GST-Ct-RBR protein incubated with reaction mixture. Coomassie Blue stained gels were used as loading control. Representative image of three independent biological replicates.

Results showed that the previously phosphorylated His-KRP4;2 by the recombinant CycD2;2-MBP–His-CDKA complex was able to inhibit kinase activity in CycD2;2–CDK complexes but using lower inhibitor concentrations (0.25 and 1.25 µg of p-His-KRP4;2; Fig. 7A); activity was no longer detected at higher concentrations (2.5 and 5.0 µg; Fig. 7C); surprisingly, the same result was obtained using CycD6;1–CDK complexes (Fig. 7B, D), particularly since a non-phosphorylated His-KRP4;2 was unable to inhibit kinase activity in these complexes (Fig. 3D).

However, His-KRP4;2 phosphorylated by the recombinant GST-CycD6;1–His-CDKA complex seemed not to affect kinase activity of immunoprecipitated CycD2;2–CDK and CycD6;1–CDK complexes when added at 0.25, 1.25 and 2.5 µg of p-His-KRP4;2 protein (Fig. 7A, B, D); inhibitory activity was only observed at the highest p-His-KRP4;2 concentration (5.0 µg; Fig 7C), but only in CycD2;2–CDK complexes; non-phosphorylated His-KRP4;2 has no inhibitory activity at the same concentration (Fig. 3A).

## Discussion

### The family of maize ICK/KRP proteins: characterization

Multiple ICK/KRPs are present in plants, and maize contains at least nine different genes. These have been divided into two classes according to a previously defined classification, five of them in class B (ICK2/KRP1;1, KRP1;2a, KRP1;2b, KRP1;3, and KRP3, specific to monocotyledonous plants), and four in class C (ICK4/KRP4;1, ICK1/KRP4;2, ICK3/KRP5;1, and KRP5;2, present in both mono- and dicotyledonous plants) and none in class A (only dicotyledonous; Torres Acosta *et al.*, 2011). The different protein motifs present in every class could be related to the function they perform in different tissues or at different physiological stages. As expected, all maize ICK/KRPs conserve motifs 1 and 2, essential for interaction with CDKs, kinase activity inhibition and binding to CycsD, both localized to the carboxyl end (CID) (Wang *et al.*, 1997; Lui *et al.*, 2000; Zhou *et al.*, 2003*a*, *b*). No information exists as to the function of the other motifs.

Half of the maize ICK/KRP proteins possess an NLS sequence (mono- or bipartite) and most of them conserve motif 7 (except KRP3), both characteristics involved in subcellular localization (Zhou *et al.*, 2003*a*;Jakoby *et al.*, 2006; Zhou *et al.*, 2006), and therefore it is very likely that maize ICK/KRPs are localized to the nucleus, as already reported for all Arabidopsis KRPs, whether they have NLS or not (Zhou *et al.*, 2006; Bird *et al.*, 2007; Torres Acosta *et al.*, 2011).

The presence of putative PEST sites in KRPs suggests degradation via the ubiquitin–proteasome system (Rogers *et al.*, 1986; Schnittger *et al.*, 2003), although this sequence by itself does not guarantee ubiquitination. Only two maize ICK/KRPs (KRP1;3 and KRP3) contain a PEST sequence; however, there is no evidence for ubiquitin-mediated degradation, or any other mechanism, of maize KRPs. In Arabidopsis, ubiquitin-dependent degradation of ICK4/KRP6 and ICK5/KRP7 seems to control male gametogenesis (Lui *et al.*, 2000; Kim *et al.*, 2008); however, only ICK4/KRP6 has a PEST site (Torres Acosta *et al.*, 2011); also, a motif in ICK1/KRP1 is critical for its stability but shows an atypical ubiquitination sequence, suggesting a degradation mechanism independent of SCF (Li *et al.*, 2016).

On the other hand, all maize ICK/KRP proteins contain CDK phosphorylation sites. In Arabidopsis ICK2/KRP2 can be phosphorylated by both CDKA;1 and CDKB1;1, modification that reduces its stability (Verkest *et al.*, 2005). Results shown in this paper also demonstrate *in vitro* maize ICK/KRP phosphorylation by CycD–CDK complexes; however, the role of this phosphorylation may be different from that suggested for Arabidopsis KRPs, as will be discussed below.

### Expression of maize ICK/KRP proteins

All maize ICK/KRP genes are expressed in different, proliferating or differentiating tissues, with different patterns, notwithstanding the class they belong to perhaps indicating that they participate both in cell cycle arrest and in cell differentiation, as has already been suggested in other plants (Wang *et al.*, 1997; Jasinski *et al.*, 2002*a*). In Arabidopsis, expression of ICK1/KRP1 and ICK2/KRP2 increases when the cell cycle is arrested (Menges *et al.*, 2005) and overexpression of ICK1/KRP1 causes endoreduplication events (Weinl *et al.*, 2005; Verkest *et al.*, 2005).

Expression of ICK/KRPs in maize during germination shows that some of them accumulate during the early hours, others are expressed at all times and all are present at 24 h of germination. Evidence indicates that during maize germination, the S phase starts by 12 h of imbibition and the first mitotic figures are observed after 24 h (Baíza *et al.*, 1989; Herrera *et al.*, 2000); thus, KRPs might be involved in both the G1–S and G2–M transitions during this developmental process. In Arabidopsis, KRP3 and KRP5 show a peak of expression in S phase, KRP4 during G2, KRP1 in the G2–M transition, KRP6 during the M–G1 transition, whereas KRP2 and KRP7 remain at constant levels during the cell cycle (Menges *et al.*, 2005).

### Inhibition of kinase activity in CycD–CDK complexes by KRPs

Members of the two different classes of maize KRPs, KRP1;1 (class B) and KRP4;2 (class C) inhibited the associated kinase activity in CycD2;2–CDK, D4;2–CDK, D5;3–CDK and D6;1–CDK complexes. KRP1;1 inhibits activity in CycD2;2–CDK, D4;2–CDK and D6;1–CDK complexes at the highest concentration used, whereas inhibition of activity in CycD5;3–CDK was achieved with a lower inhibitor concentration. Since all CycsD studied here bind A or B type CDKs (Godínez-Palma *et al.*, 2013; Vázquez-Ramos, unpublished data) the kinase activity measured must be due to complexes containing either CDK. CycD5;3–CDK seems to be more sensitive to KRP inhibition and, incidentally, CycD5;3 binds mainly to CDKA during early maize germination (Godínez-Palma *et al.*, 2013); this suggests that this higher sensitivity might be due to preferential association to only one and not both types of CDKs.

Similarly, kinase activity inhibition by His-KRP4;2 was only visible at the highest concentration for CDK complexes containing CycsD2;2, D4;2 and D5;3; however, activity in CycD6;1–CDK complexes was not inhibited at any concentration used. CycD6;1 is peculiar because it lacks the LXCXE motif, a characteristic sequence in all CycsD, related to retinoblastoma-related (RBR) protein binding. It has been demonstrated for Arabidopsis and maize CycD6;1 proteins that this motif is not necessary for RBR phosphorylation (Cruz-Ramírez, *et al.*, 2012; Zamora-Zaragoza, 2015, unpublished data). Thus, our results suggest that the difference of kinase activity inhibition by maize ICK/KRPs might be due to a different KRP association capacity to CycD–CDK complexes. CycD2;2-MBP binds GST-CDKA, His-KRP1;1 and His-KRP4;2, while PeX-CycD6;1 binds GST-CDKA and His-KRP1;1 but not His-KRP4;2, and this could determine the degree of inhibitory activity; i.e. inhibition may require KRP binding to both the cyclin and the CDK proteins.

It is puzzling that kinase activity inhibition for most CycD–CDK complexes is achieved when KRP concentration is just doubled (from 2.5 to 5 µg); in fact, addition of 3.75 µg already shows some inhibition (data not shown). We do not know if this is due to a certain stoichiometric relationship or if it is due to some structural modification of KRPs, perhaps a structural change due to a gradual KRP phosphorylation that finally makes it a more potent inhibitor (see below).

Preliminary results suggest that maize CycD6;1 binds Zeama-RBR in different sites (Zamora-Zaragoza, unpublished data); perhaps this difference of CycD6;1 association with substrates, compared with the association characteristics of canonical cyclins D, could explain the difference in interaction between PeX-CycD6;1 and the two KRPs: CycD6;1 may bind other sites than the CID in KRPs, perhaps a sequence present in the amino region of His-KRP1;1, which is absent in the shorter His-KRP4;2 (see Fig. 1). In tomato KRP1, there appears to be a sequence in the central region of the protein that is bound by CycD3 (Nafati *et al.*, 2010).

### Phosphorylation of KRPs modify their inhibitory capacity

All maize ICK/KRPs have at least one putative CDK phosphorylation site and results demonstrated that all CycD–CDK complexes used here phosphorylated recombinant His-KRP1;1 and His-KRP4;2 proteins, besides phosphorylating Zeama-GST-Ct-RBR (Fig 5). In Arabidopsis ICK/KRPs and also in yeast Sic1p protein, phosphorylation by Cyc–CDK complexes promotes protein degradation, an essential mechanism for control and progression of the cell cycle (Nishizawa *et al.*, 1998; Verkest *et al.*, 2005). In this context, that maize KRPs are phosphorylated is not surprising.

What is relevant in maize KRP phosphorylation is that the inhibitory activity can be selectively enhanced. His-KRP4;2 phosphorylation by the different recombinant kinase complexes has a differential effect on kinase activity in immunoprecipitated CycD–CDK complexes from embryo axes. His-KRP4;2 phosphorylation by a recombinant CycD2;2-MBP–His-CDKA complex increases kinase activity inhibition of CycD–CDK complexes, even at lower KRP concentrations. Recombinant alfalfa KRP is phosphorylated by a calmodulin-dependent kinase, increasing KRP inhibitory activity on MtCycA;2–CDKA;1 (Pettkó-Szandtner *et al.*, 2006). There is no information, to our knowledge, that in alfalfa, or any other plant, Cyc–CDK complex phosphorylates and activates KRPs.

On the other hand, His-KRP4;2 phosphorylated by a recombinant GST-CycD6;1–His-CDKA complex behaves differently, having little, or no inhibitory activity against CycD–CDK complexes at all concentrations tested. These results suggest that the different CycD–CDK complexes phosphorylate His-KRP4;2 in different residues, and depending on which is phosphorylated, the inhibitory capacity of His-KRP4;2 could be enhanced or reduced. In this context, it is relevant that phosphorylated KRPs can be re-phosphorylated by another CycD–CDK, as indicated in Fig. 6, giving evidence that more than one residue could be modified.

A differential phosphorylation of His-KRP4;2 could be the result of the direct or indirect interaction of this KRP with CycsD; interaction of CycD2;2-MBP and His-KRP4;2 could result in His-CDKA phosphorylation of canonical residues, whereas the indirect interaction of CycD6;1 and His-KRP4;2 could produce phosphorylation in other residues, which modify the inhibitory capacity of His-KRP4;2.

Work with budding yeast has demonstrated that Sic1p displays a differential binding affinity toward G1 cyclins (CLNs) and S-M cyclins (CLBs) (Kõivomägi *et al.*, 2011). CDK1–CLNs bind but are not inhibited by Sic1p; instead Sic1p is phosphorylated at multiple sites targeting it to destruction (Cross *et al.*, 2007), which in turn causes the release of the CLB5–CDK1 complex from Sic1p inhibitory activity, thus triggering S phase onset (Verma *et al.*, 1997; Nash *et al.*, 2001). In maize, all cyclins D studied are present through the whole cell cycle process that takes place during germination (De Jesús Juárez *et al.*, 2008; Lara-Núñez *et al.*, 2008; Godínez-Palma *et al.*, 2013; Vázquez-Ramos, unpublished data). In this context, it is very interesting that the CycD6;1–CDK complex is not inhibited by KRP4;2 but does phosphorylate it, somehow resembling the situation in budding yeast, perhaps establishing a role for this kinase complex in promoting cell cycle advance. Therefore, it is tempting to speculate that phosphorylation of KRPs in different residues, or by different CycD–CDK complexes, would be responsible for their association, activation, inhibition or destruction. This could be part of the mechanism by which cell cycle transitions are controlled and would depend on the appearance of the different CycD–CDKs.

It will be very important to understand which residues are phosphorylated in every KRP and if phosphorylation of other residues modifies their stability or inhibitory capacity. Finally, the great variety of KRPs and also of CycD–CDK complexes point to a complex regulatory network that perhaps is related to the developmental needs of different tissues or responds to the different environmental conditions to which plants have to adapt.

## Supplementary data

Supplementary data are available at *JXB* online.

Fig. S1. Alignment of KRP1;2a and KRP1;2b genes and identity of maize and rice ICK/KRP genes.

Fig. S2. Identification of motifs and domains in maize ICK/KRP genes.

Fig. S3. Validation of anti-CycD6;1 antibodies.

Fig. S4. Kinase activity controls.

Fig. S5. Negative controls of interaction of His-KRP proteins and resins (complete figure).

Fig. S6. Interaction assays among CycD2;2, CycD6;1, CDKA, KRP1;1 and KRP4;2 (complete figure)

Fig. S7. Interaction CycDs–CDKA–KRPs (complete figure).

Fig. S8. Controls for purification of phosphorylated KRP4;2.

Fig. S9. No phosphorylacion of p-His-KRP4;2^(CycD2;2-MBP/His-CDKA or GST-CycD6;1/His-CDKA)^ by recombinant CycD2;2-MBP–His-CDKA and GST-CycD6;1–His-CDKA complexes

Table S1. Reported access numbers for *Arabidopsis thaliana* ICK/KRP and *Orysa sativa*.

Table S2. Access numbers in Ensembl plants database; http://plants.ensembl.org;Kersey *et al.* (2016).

Table S3. Primers and experimental conditions for expression of maize ICK/KRP genes using semiquantitative RT-PCR.

Table S4. Conditions for recombinant protein production.

## Supplementary Material

Supplementary_tables_S1_S4_Figures_S1_S9
