# Supplementary material for: Two maize Kip-related proteins differentially interact with, inhibit and are phosphorylated by cyclin D–cyclin-dependent kinase complexes
Source: J Exp Bot. 2017 Mar 24;68(7):1585–97. doi: 10.1093/jxb/erx054 (PMC5444471; doi:10.1093/jxb/erx054)
Supplement: Supplementary_tables_S1_S4_Figures_S1_S9 [file erx054_suppl_Supplementary_tables_S1_S4_Figures_S1_S9.pdf]

Plant	Name	Accsession number	Resource
Arabidopsis thaliana	Arath; ICK1/KRP1	Q67Y93	TAIR
	Arath; ICK2/KRP2	Q9SCR2	TAIR
	Arath; ICK3/KRP5	Q9LRY0	TAIR
	Arath; ICK4/KRP6	Q0WNX9	TAIR
	Arath; ICK5/KRP7	Q94CL9	TAIR
	Arath; ICK6/KRP3	Q9FKB5	TAIR
	Arath; ICK7/KRP4	Q8GYJ3	TAIR
Oryza sativa (*subesp. Japonica*)	Orysa;KRP1	ABB70058	NCBI
	Orysa;KRP2	ABB70059	NCBI
	Orysa;KRP3	ABB70060	NCBI
	Orysa;KRP4	ABB70061	NCBI
	Orysa;KRP5	ABB70062	NCBI
	Orysa;KRP6	ACT53244	NCBI

Table. S1

Supplementary Table 1. Previously reported access numbers for *Arabidopsis thaliana* ICK/KRP and *Orysa sativa* (NCBI- National Center Biotechnology Information; http://www.ncbi.nim.nih.gov). The Arabidopsis Information Resourse (TAIR), database of genetic and molecular biology data for the model higher plant *Arabidopsis thaliana*.

ICK/KRP	Gene MaizeSeq Accession number	Clone	Chromosomal location	Translation length
ICK2/KRP1;1	GRMZM2G101613_T01	AC210721.4-Contig34	Chromosome 5: 209,721,762-209.723,5 07 forward strand	253
KRP1;2a	GRMZM2G084570_T01	AC1839143.3-Contig55	Chromosome 4: 178, 744, 162-178,754,8 97 reverse strand	263
KRP1;2b	GRMZM5G854731_T01	AC202932.3-Contig62	Chromosome 8:112,022,205-112,023,940 Forward strand	263
KRP1;3	GRMZM2G343769_T01	AC209339.3-Contig23	Chromosome 9: 7,869,191-7,870,616 forward strand	275
KRP3	GRMZM2G154414_T01	AC210175.3-Contig24	Chromosome 4: 5,405,531-5,406,955 reverse strand	216
ICK1/KRP4;2	GRMZM2G037926_T01	AC210175.3-Contig25	Chromosome 5: 29,621,966-29,625,374 Reverse strand	192
ICK4/KRP4;1	GRMZM2G116885_T01	AC185516.3-Contig28	Chromosome 1: 232,918,113-232,921,288 forward strand	190
ICK3/KRP5;1	GRMZM2G358931_T01	AC194161.3-Contig105	Chromosome 9: 151,985,979-151,989,464 forward strand	215
KRP5;2	GRMZM2G157510_T01	AC1944863.3-Contig103	Chromosome 1: 8,447,465-8,451,013 forward strand	213

Table S2.

Supplementary Table 2. Access numbers in Ensembl plants database; http://plants.ensembl.org; Kersey *et al.,* 2016, chromosomal location and size of genes corresponding to maize ICK/KRPs.

	Primer sequense (5´-3´)	Fragment (bp)	RT-PCR Reaction conditions in germinating seeds and plant tissues
**Zeama;KRP1;1**	FW CGC ATG CTG TTC ATG GCA C	688	2 min at 94°C
	RV AAG CAA GGC ATT TGG ACG AG		35 cycles (30 sec at 94°C, 30 sec at 58.8°C, 2 min at 72°C)
**Zeama;KRP1;2a Zeama;KRP1;2b**	FW GCA AGC GAG AAG AAG GCA GT	1020	2 min at 94°C
	RV CGC TGT CAT TCT ACA AGT CTA CTC C		35 cycles (30 sec at 94°C, 30 sec at 60.4°C, 2 min at 72°C)
**Zeama;KRP1;3**	FW CCA GGC ACG GGT AGA TCG TC	402	2 min at 94°C
	RV TTT ATC GTC TGT ACG CTC GTC CCA		35 cycles (30 sec at 94°C, 30 sec at 58.8°C, 2 min at 72°C)
**Zeama;KRP3**	FW TTC GCA GAG GCG TAC AAT TAC GAC	318	2 min at 94°C
	RV GCA CCA TCA GCT GCA TAT ACG CTA		35 cycles (30 sec at 94°C, 30 sec at 60.4°C, 2 min at 72°C)
**Zeama;KRP4;1**	FW GTC CCG ACC TCT TCT CTC CTA	591	2 min at 94°C
	RV TGG GAA GAG TGG CTG GAT CTT GTT		35 cycles (30 sec at 94°C, 30 sec at 60.4°C, 2 min at 72°C)
**Zeama;KRP4;2**	FW AGA CGA AGT GGG AGG AAG GTG CT	503	2 min at 94°C
	RV AGC GGG GAG TGA GTT CCA TTC CAT		35 cycles (30 sec at 94°C, 30 sec at 60.4°C, 2 min at 72°C)
**Zeama;KRP5;1**	FW TCG CAG GAT GGA AAC CTC AGT C	439	2 min at 94°C
	RV TAG GCC TAC GCT TGA GAT GGA T		35 cycles (30 sec at 94°C, 30 sec at 60.4°C, 2 min at 72°C)
**Zeama;KRP5;2**	FW TTA TTC CCA GGA TTC CCC TTG CGA	628	2 min at 94°C
	RV CTC GGT ATG AAC CGG CAG CTC		35 cycles (30 sec at 94°C, 30 sec at 58.8°C, 2 min at 72°C)
**18 S**	FW TCG AAC TGT GAA ACT GCG AAT GG	387	2 min at 94°C
	RV CCG TGT CAG GAT TGG GTA ATT TGC		35 cycles (30 sec at 94°C, 30 sec at 57°C, 2 min at 72°C)

Table S3

Supplementary Table S3. Primers and experimental conditions for expression of maize ICK/KRP genes using semiquantitative RT-PCR.

Protein	Strain	Vector	Molecular mass (kDa)	Antibiotic resistance	Purification resin
GST	*E. coli* BL21-CodonPLus-RIL	pGEX-4T2	25	Ampicillin Chloramphenicol	Glutathione Agarose Resin
GST-CDKA			60		
GST-CycD6;1	*E. coli* BL21		62		
PeX-CycD6;1	*E. coli* BL21-CodonPlus-RIL	pPal7 (SSP)	41	Ampicillin Cloranphenicol	Profinity eXact Purification Resin
MBP		pMAL-c5X (MBP; Maltose binding protein)	40	Ampicillin Chloramphenicol	Amylose Resin
CycD2;2-MBP		pCDFDuet-1	72	Streptomycin Chloramphenicol	
His-CDKA		pProex HTb	34	Ampicillin Chloramphenicol	Ni-NTA Agarose
His-KRP1;1			25		
His-KRP4;2			29		

Table S4

Supplementary Table S4. Conditions for recombinant protein production. Gen, Strain, Vector, Molecular mass, Antibiotic resistance and Purification resin used.

A)

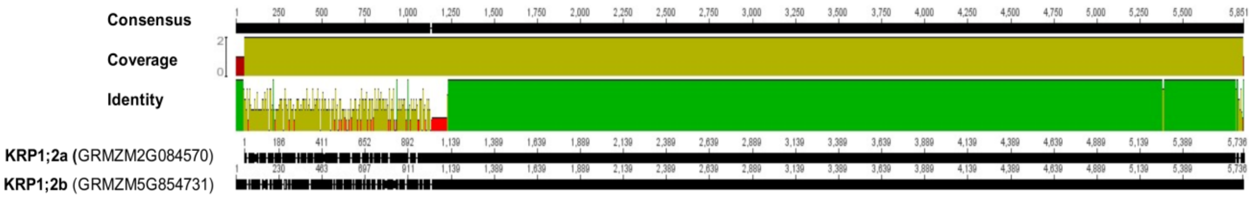

B)

Specie	Zeama; KRP1;1/ICK2	Zeama; KRP1;2a KRP1;2b	Zeama; KRP1;3	Zeama; KRP3	Zeama; KRP4;1	Zeama; KRP4;2/ICK1	Zeama; KRP5;1	Zeama; KRP5;2/ICK3
**Oryza;KRP1**	**58.5%**	**57.0%**	**38.7%**	15.7%	18.9%	18.6%	20.6%	20.9%
**Oryza;KRP2**	25.2%	23.7%	24.4%	17.3%	15.9%	14.1%	16.9%	16.9%
**Oryza;KRP3**	17.2%	17.3%	19.4%	**31.8%**	11.4%	11.9%	12.7%	12.7%
**Oryza;KRP4**	20.2%	18.5%	19.8%	15.8%	**62.7%**	**60.2%**	50.6%	49.7%
**Oryza;KRP5**	23.5%	21.9%	20.8%	12.4%	52.2%	49.5%	**69.7%**	**68.5%**
**Oryza;KRP6**	11.4%	10.2%	9.8%	11.5%	7.8%	6.9%	8.1%	8.1%
**Zeama;KRP1;1/ICK2**	ID	**73.9%**	37.4%	12.6%	20.8%	20.5%	22.3%	21.1%
**Zeama;KRP1;2a Zeama;KRP1;2b**	**73.9%**	ID	36.8%	14.0%	20.5%	19.4%	21.0%	20.0%
**Zeama;KRP1;3**	37.4%	36.8%	ID	14.6%	18.4%	18.4%	19.3%	18.4%
**Zeama;KRP3**	12.6%	14.0%	14.6%	ID	15.9%	15.9%	13.1%	14.3%
**Zeama;KRP4;1/ICK4**	20.8%	20.5%	18.4%	15.9%	ID	**82.8%**	52.0%	51.3%
**Zeama;KRP4;2/ICK1**	20.5%	19.4%	18.4%	15.9%	**82.8%**	ID	50.2%	49.5%
**Zeama;KRP5;1**	22.3%	21.0%	19.3%	13.1%	52.0%	50.2%	ID	**88.4%**
**Zeama;KRP5;2/ICK3**	21.1%	20.0%	18.4%	14.3%	51.3%	49.5%	**88.4%**	ID

Figure S1

Supplementary Figure S1. Alignment of KRP1;2a and KRP1;2b genes and identity of maize and rice ICK/KRP genes. **A)** Comparison and alignment of KRP1;2a y KRP1;2b genes, Green bar indicates nucleotide identity in the coding region and differences after 3000 nucleotides upstream the 5´UTR. **B)** Percentage of identity matrix between protein sequences in maize and rice.

A)

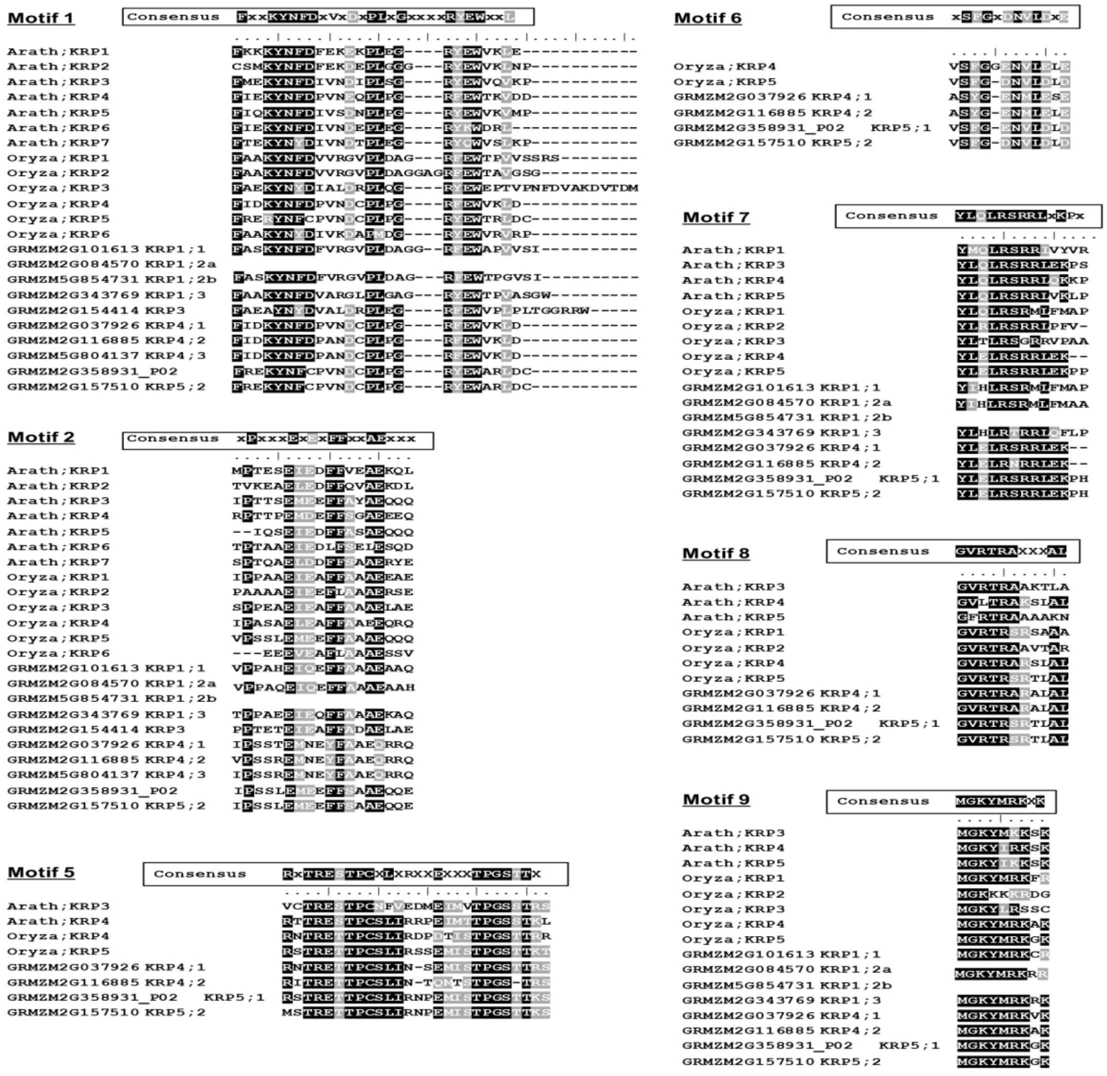

B)

ICK/KRP	NLS monopartite (aa, score)	NLS Bipartite (aa, score)	Potential PEST box (aa, score)	Poor PEST box (aa, score)	Putative CDK phosphorylation sites (aa)
KRP1;1	---	46 – 75 (5.0)	---	76 - 114 (-8.50), 50 - 72 (-15.34), 188 - 208 (-7.08), 145 – 162 (-10.05)	T92, S117,T168, S171, T197
KRP1;2a KRP1;2b	---	5 - 33 (5.9)	---	87 - 124 (-19.21), 197 - 232 (-2.65), 51 - 82 (-15.21), 152- 168 (-4.60), 140-152 (0.65)	T102, S127, T173, T174, S202, S203, T258
KRP1;3	4 - 13 (12.0)	---	187 - 203 (7.28)	118-140 (-9.00), 26 -45 (-14.78), 101 - 118 (1.12), 224-241 (-4.81)	T16, S121, T178, S181, T226, T269
KRP3	---	---	145 - 182 (7.73)	31 - 44 (-4.30), 202 - 214 (-20.56)	S11, S42, S71, S85, T154, S156, S158, S160
KRP4;1	---	---	---	78 - 106 (-4.55), 109 - 130 (4.63), 9 - 27 (-22.80), 145 - 162 (-6.71)	T112, S123, T124
KRP4;2	---	---	---	108 - 128 (4.05), 9-27 (-22.80)	T111, S122, T123
KRP5;1	---	5 - 34 (5.2)	---	167 - 188 (-3.58), 9 - 28 (-24.74), 135 - 148 (2.18)	T129, S141, T142, T151, S152, S154
KRP5;2	---	---	---	165-186 (-3.58), 9 - 28 (-25.29)	S72, T125, S137, T138, S141, T147, S148, S149, T161

Figure S2

Supplementary Figure S2. Identification of motifs and domains in maize ICK/KRP genes. **A)** Identification and alignment of the characteristic motifs in ICK/KRP genes from Arabidopsis, rice and maize. **Motif 1;** Binding consensus sequence with CDKs. **Motif 2**; Binding consensus sequence with Cyclins D. **B)** Localization of specific domains in maize ICK/KPRs. Domains 1 and 2 for CDKs and Cyclins D binding; mono or bipartite nuclear localization signal (NLS); localization of PEST boxes and putative CDK phosphorylation sites.

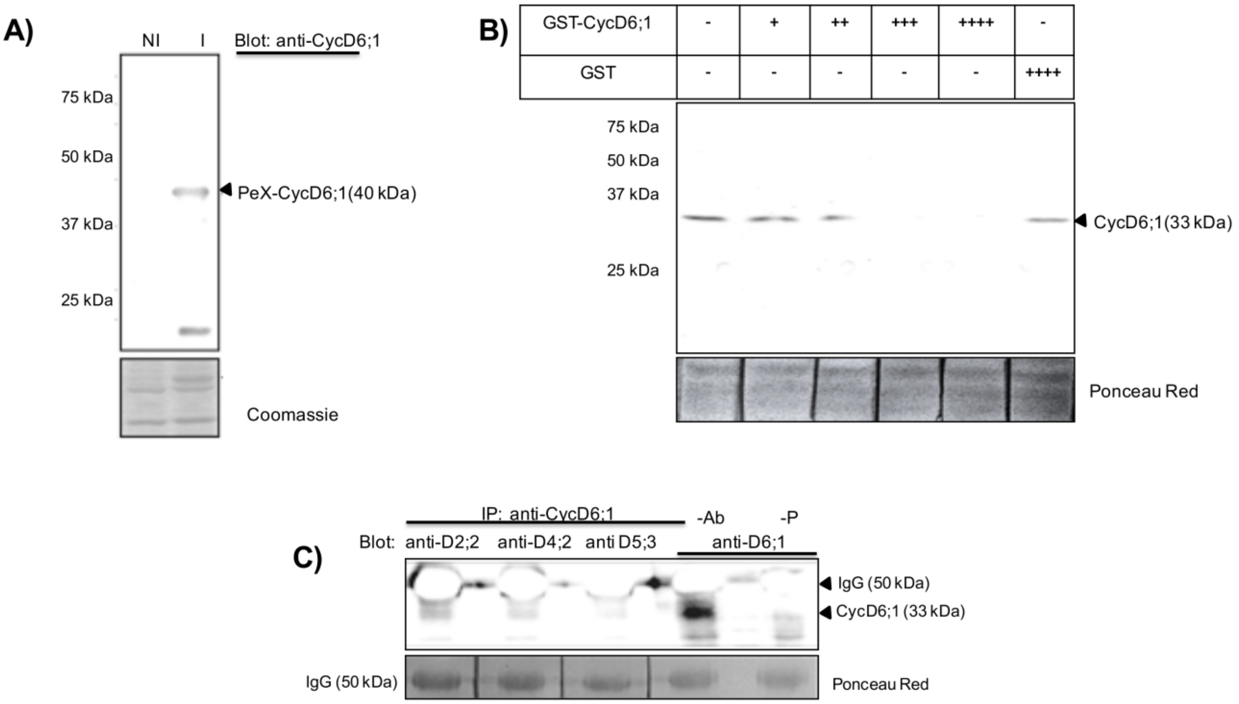

Figure S3

Supplementary Figure S3. Validation of anti-CycD6;1 antibodies. **A)** An overexpressed full length CycD6;1 fused to Profinity eXact Tag (PeX:CycD6;1) was specifically recognized by purified anti-CycD6;1 antibodies in bacterial extracts only after induction with 0.2 mM IPTG. **B)** Immunocompetition assay. Anti-CycD6;1 antibodies were pre incubated with 0, 5, 20, 60 and 100 µg of GST:CycD6;1-Ag and then used to immunodetect endogenous CycD6;1 in protein extracts from embryo axes. GST (100 µg) was used as negative competitor. **C)** Anti-CycD6;1 immunoprecipitated proteins from embryo axes protein extracts, assays were loaded in a SDS-PAGE and blotted with anti-CycD2;2, anti-CycD4;2, anti-CycD5;3 and anti-CycD6;1 antibodies. Negative controls, immunoprecipitation assays were performed without antibodies (-Ab) or without protein extracts (-P). NI, Not Induced; I, Induced.

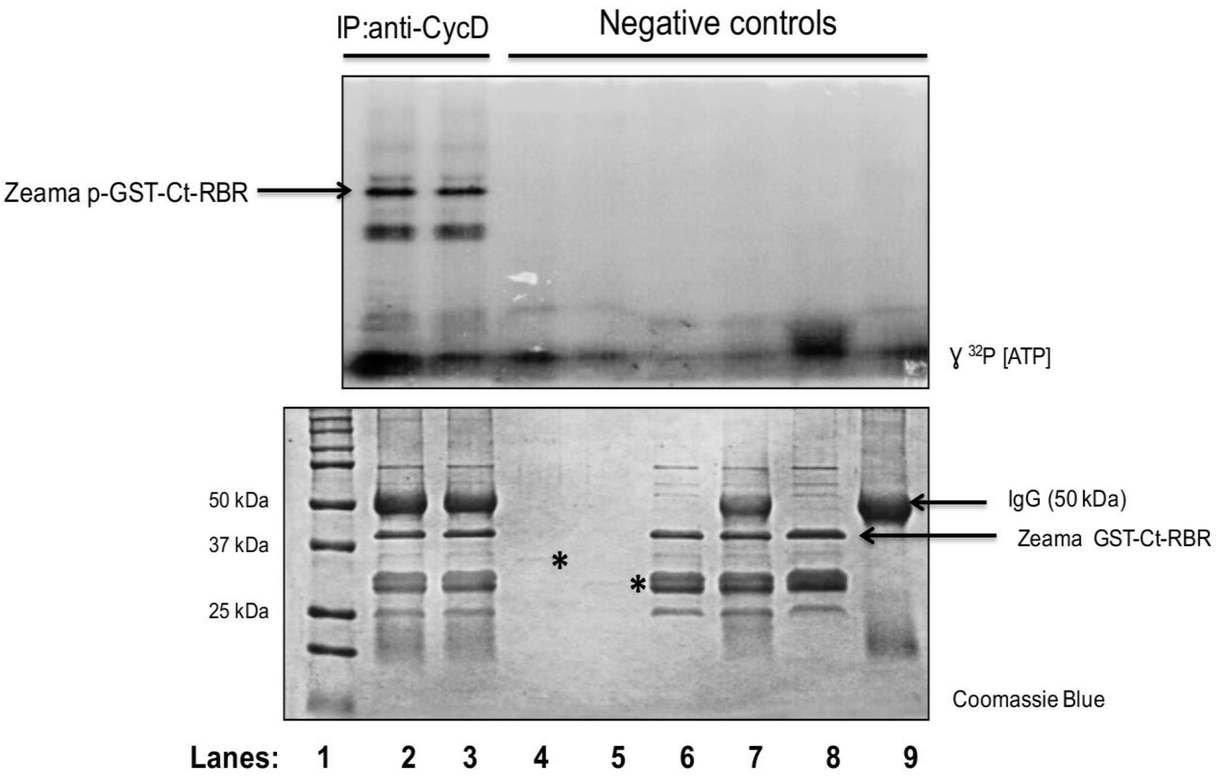

Figure S4

Supplementary Figure S4. Kinase activity controls. **Lane 1**; Protein molecular mass markers. **Lane 2;** Kinase activity of CycD2;2-CDK complexes on Zeama GST-Ct-RBR. **Lane 3;** Kinase activity of CycD2;2-CDK complexes previously incubated with 5.0 µg BSA, on Zeama GST-Ct-RBR. **Lane 4;** Recombinant His-KRP1;1 protein, kinase buffer and ^{32}P [ATP]. **Lane 5;** Recombinant His-KRP4;2 protein, kinase buffer and ^{32}P [ATP]. **Lane 6;** Agarose A incubated with protein extracts, Zeama GST-Ct-RBR, kinase buffer and ^{32}P [ATP]. **Lane 7;** Agarose A incubated with anti-CycD2;2 antibody, Zeama Ct-RBR, kinase buffer, ^{32}P [ATP], without protein extracts. **Lane 8;** Zeama GST-Ct-RBR, kinase buffer and ^{32}P [ATP]. **Lane 9;** Anti-CycD2;2 immunoprecipitation, no Zeama GST-Ct-RBR added. (*) Purified recombinant His-KRP1;1 and His-KRP4;2 proteins. Coomassie blue stained gel was used as loading control. Representative image of three independent biological replicates.

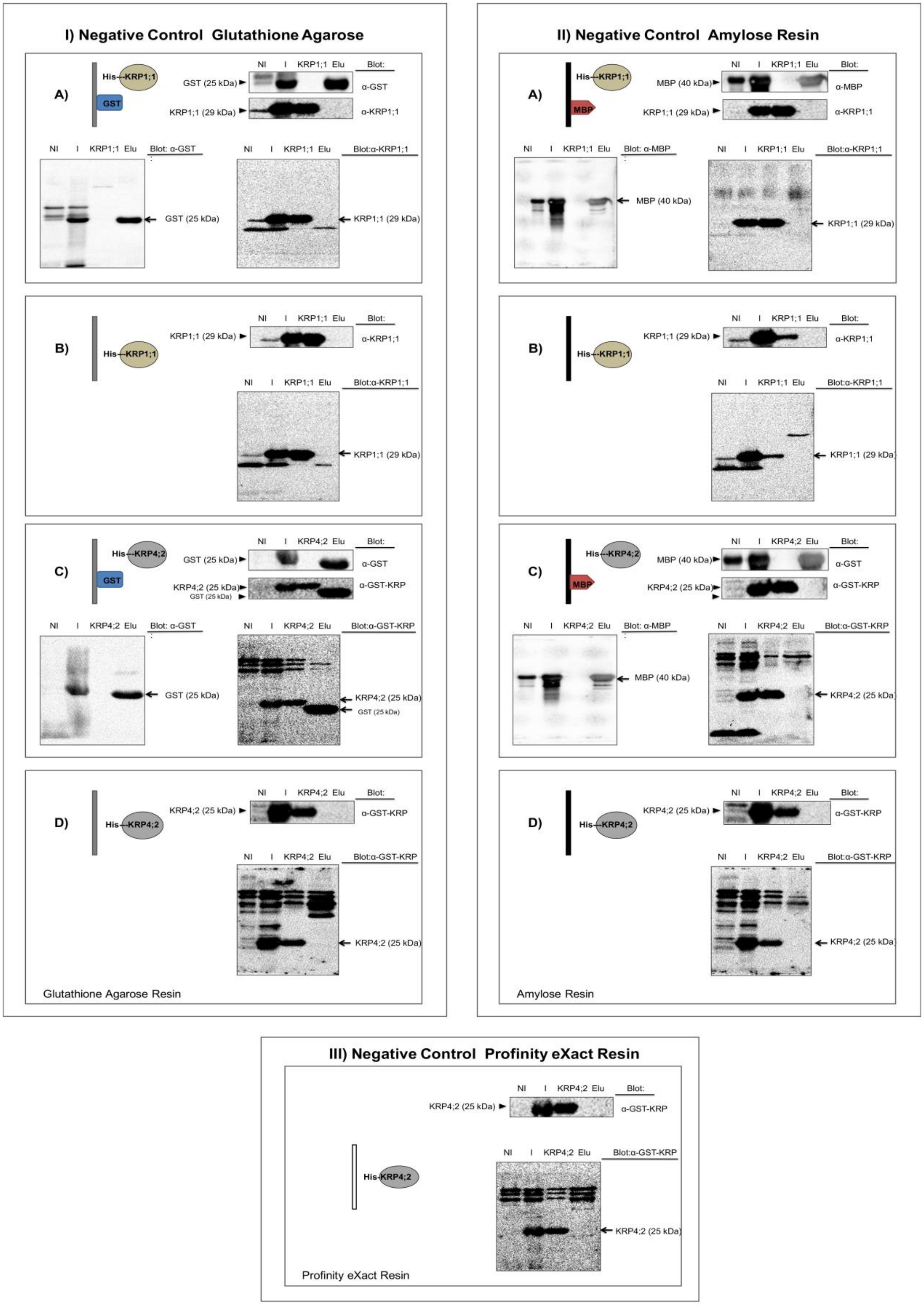
I) Negative Control Glutathione Agarose
A)
His-KRP1;1
GST
NI I KRP1;1 Elu Blot:
GST (25 kDa)
α-GST
KRP1;1 (29 kDa)
α-KRP1;1
Blot: α-GST
Blot:α-KRP1;1
B)
C)
His-KRP4;2
KRP4;2 (25 kDa)
α-GST-KRP
Blot:α-GST-KRP
D)
Glutathione Agarose Resin
II) Negative Control Amylose Resin
MBP
MBP (40 kDa)
α-MBP
Blot: α-MBP
Amylose Resin
III) Negative Control Profinity eXact Resin
Profinity eXact Resin

Figure S5

Supplementary Figure S5. Negative controls of interaction of His-KRP proteins and resins (Complete figure). **I)** No interaction between KRP recombinant proteins and Glutathione-Agarose Resin: **A)** No interaction between GST protein and His-KRP1;1, GST recognition (left membrane), no KRP1;1 recognition (right membrane) **. B)** No interaction between His-KRP1;1 protein and Glutathione-Agarose resin. **C)** No interaction between GST protein and His-KRP4;2, GST recognition (left membrane), no KRP recognition (right membrane). **D)** No interaction between His-KRP4;2 protein and Glutathione-Agarose Resin. **II)** Negative controls of interaction between recombinant proteins and Amylose Resin. **A)** No interaction between MBP protein and His-KRP1;1, MBP recognition (left membrane), no KRP1;1 recognition (right membrane). **B)** No interaction between His-KRP1;1 protein and Amylose Resin. **C)** No interaction between MBP protein and His-KRP4;2, MBP recognition (left membrane), no KRP4;2 recognition (right membrane). **D)** No interaction between His-KRP4;2 protein and Amylose Resin. **III)** No interaction between His-KRP4;2 protein and Profinity eXact Resin. Western Blots were performed using anti-MBP, anti-KRP1;1, anti-KRP or anti-CycD6;1 antibodies. **NI,** Not Induced (No IPTG added). **I**, Induced (IPTG added for protein induction). **KRP1;1:** purified His-KRP1;1 (added as positive control for KRP identification). **KRP4;2:** purified His-KRP4;2 (added as positive control for KRP identification). **Elu,** Elution of resin-bound proteins. Representative image of three independent biological replicates.

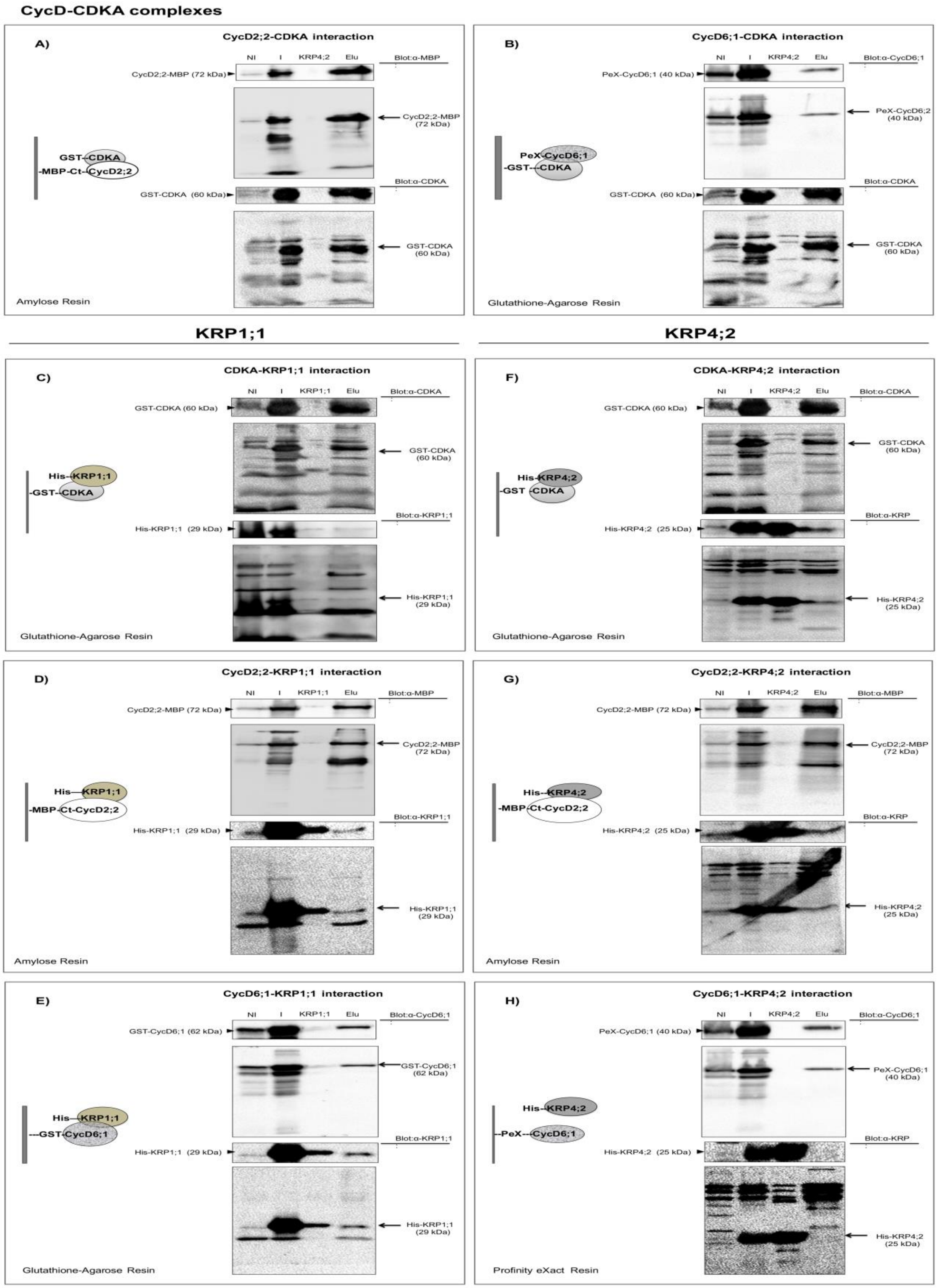
CycD-CDKA complexes
A)
CycD2;2-CDKA interaction
NI
I
KRP4;2
Elu
Blot:α-MBP
CycD2;2-MBP (72 kDa)
CycD2;2-MBP (72 kDa)
GST-CDKA
-MBP-Ct-CycD2;2
Blot:α-CDKA
GST-CDKA (60 kDa)
GST-CDKA (60 kDa)
Amylose Resin
B)
CycD6;1-CDKA interaction
NI
I
KRP4;2
Elu
Blot:α-CycD6;1
PeX-CycD6;1 (40 kDa)
PeX-CycD6;2 (40 kDa)
PeX-CycD6;1
-GST-CDKA
Blot:α-CDKA
GST-CDKA (60 kDa)
GST-CDKA (60 kDa)
Glutathione-Agarose Resin
KRP1;1
KRP4;2
C)
CDKA-KRP1;1 interaction
NI
I
KRP1;1
Elu
Blot:α-CDKA
GST-CDKA (60 kDa)
GST-CDKA (60 kDa)
His-KRP1;1
-GST-CDKA
Blot:α-KRP1;1
His-KRP1;1 (29 kDa)
His-KRP1;1 (29 kDa)
Glutathione-Agarose Resin
F)
CDKA-KRP4;2 interaction
NI
I
KRP4;2
Elu
Blot:α-CDKA
GST-CDKA (60 kDa)
GST-CDKA (60 kDa)
His-KRP4;2
-GST-CDKA
Blot:α-KRP
His-KRP4;2 (25 kDa)
His-KRP4;2 (25 kDa)
Glutathione-Agarose Resin
D)
CycD2;2-KRP1;1 interaction
NI
I
KRP1;1
Elu
Blot:α-MBP
CycD2;2-MBP (72 kDa)
CycD2;2-MBP (72 kDa)
His-KRP1;1
-MBP-Ct-CycD2;2
Blot:α-KRP1;1
His-KRP1;1 (29 kDa)
His-KRP1;1 (29 kDa)
Amylose Resin
G)
CycD2;2-KRP4;2 interaction
NI
I
KRP4;2
Elu
Blot:α-MBP
CycD2;2-MBP (72 kDa)
CycD2;2-MBP (72 kDa)
His-KRP4;2
-MBP-Ct-CycD2;2
Blot:α-KRP
His-KRP4;2 (25 kDa)
His-KRP4;2 (25 kDa)
Amylose Resin
E)
CycD6;1-KRP1;1 interaction
NI
I
KRP1;1
Elu
Blot:α-CycD6;1
GST-CycD6;1 (62 kDa)
GST-CycD6;1 (62 kDa)
His-KRP1;1
---GST-CycD6;1
Blot:α-KRP1;1
His-KRP1;1 (29 kDa)
His-KRP1;1 (29 kDa)
Glutathione-Agarose Resin
H)
CycD6;1-KRP4;2 interaction
NI
I
KRP4;2
Elu
Blot:α-CycD6;1
PeX-CycD6;1 (40 kDa)
PeX-CycD6;1 (40 kDa)
His-KRP4;2
--PeX--CycD6;1
Blot:α-KRP
His-KRP4;2 (25 kDa)
His-KRP4;2 (25 kDa)
Profinity eXact Resin

Figure S6.

Supplementary Figure S6. Interaction assays among CycD2;2, CycD6;1, CDKA, KRP1;1 and KRP4;2 (complete figure). **A)** Not induced **(NI)** and Induced **(I)**, CycD2;2-MBP (upper membrane), GST-CDKA (lower membrane) proteins, no recognition of His-KRP4;2 protein by anti-MBP or anti-CDK antibodies. Interaction of CycD2;2 and CDKA as shown by immunodetection of both proteins after Elution (Elu) using CycD2;2-MBP as resin-bound protein**.** **B)** same as **A)** but using PeX-CycD6;1 (upper membrane) and GST-CDKA (lower membrane), no recognition of His-KRP4;2 protein by anti-CycD6;1 or anti-CDKA antibodies. Interaction of CycD6;1 and CDKA as shown by immunodetection of both proteins after Elution (Elu) using GST-CDKA as resin-bound protein.**C-H) I**nteractions between pairs of proteins: **C)** GST-CDKA-His-KRP1;1, using Glutathione-agarose resin; **D)** MBP-Ct-CycD2;2-His-KRP1;1, using Amylose Resin. **E)** GST-CycD6;1-His-KRP1:1 using Glutathione-Agarose Resin. **F)** GST-CDKA-His-KRP4;2, using Glutathione-Agarose Resin. **G)** MBP-Ct-CycD2;1-His-KRP4;2, using Amylose Resin. **H)** Pex-CycD6;1-His-KRP4;2, using Profinity eXact resin. In all cases, the first protein in the pair is shown in the upper membrane and the second protein of the pair in the lower membrane, Not induced **(NI),** or Induced **(I).** Immunorecognition of either **KRP1;1** (**C, D and E**) or **KRP4;2** (**F, G and H**) was used in the lane “KRP” as a negative control (use of the antibody against the partner protein) or positive control (use of the corresponding KRP antibody). Representative image of three independent biological replicates.

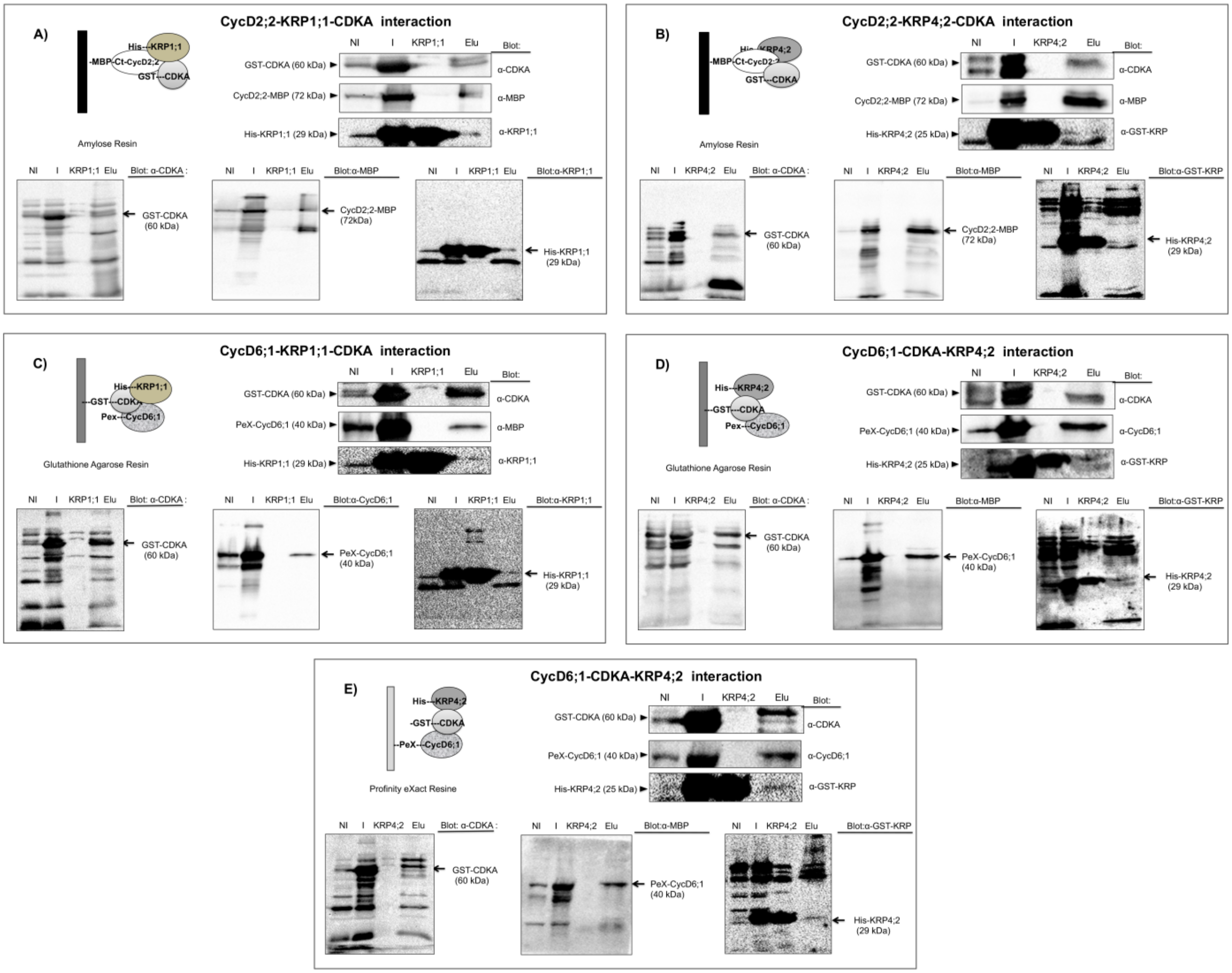
A)
CycD2;2-KRP1;1-CDKA interaction
Amylose Resin
B)
CycD2;2-KRP4;2-CDKA interaction
C)
CycD6;1-KRP1;1-CDKA interaction
Glutathione Agarose Resin
D)
CycD6;1-CDKA-KRP4;2 interaction
E)
CycD6;1-CDKA-KRP4;2 interaction
Profinity eXact Resine

Figure S7.

Supplementary Figure S7. Expression and *in vitro* interaction assays between CycD2;2-CDKA-KRP1;1 or KRP4;2 and CycD6;1-CDKA-KRP1;1 or KRP4;2 using different resins. Complete figures of each immunodetection are shown. **A)** Interaction between CycD2;2-CDKA-KRP1;1 proteins, immunorecognition after elution of GST-CDKA (left membrane), CycD2;2-MBP (middle membrane) and His-KRP1;1 (right membrane derecha), amylose resin was used. **B)** Interaction between CycD2;2-CDKA-KRP4;2 proteins, immunorecognition after elution of GST-CDKA (left membrane), CycD2;2-MBP (middle membrane) and His-KRP4;2 (right membrane), Glutathione agarose resin was used. **C)** Interaction between CycD6;1-CDKA-KRP1;1 proteins, immunorecognition after elution of GST-CDKA (left membrane), PeX-CycD6;1 (middle membrane) and His-KRP1;1 (right membrane), Profinity eXact resin was used. **D)** Interaction between CycD6;1-CDKA-KRP4;2 proteins, immunorecognition after elution of GST-CDKA (left membrane), PeX-CycD6;1 (middle membrane) and His-KRP4;2 (right membrane), Glutathione agarose resin was used. **E)** Interaction between CycD6;1-CDKA-KRP4;2 proteins, immunorecognition after elution of GST-CDKA (left membrane), PeX-CycD6;1 (middle membrane) and His-KRP4;2 (right membrane), Profinity eXact resin was used. **NI**, Not induced (no IPTG added), **I,** induced (IPTG added to induce proteins). **Elu,** Elution of resin-bound proteins. Western Blots were performed using antibodies against GST, MBP, KRP1;1, GST-KRP or CycD6;1. Representative image of three independent biological replicates.

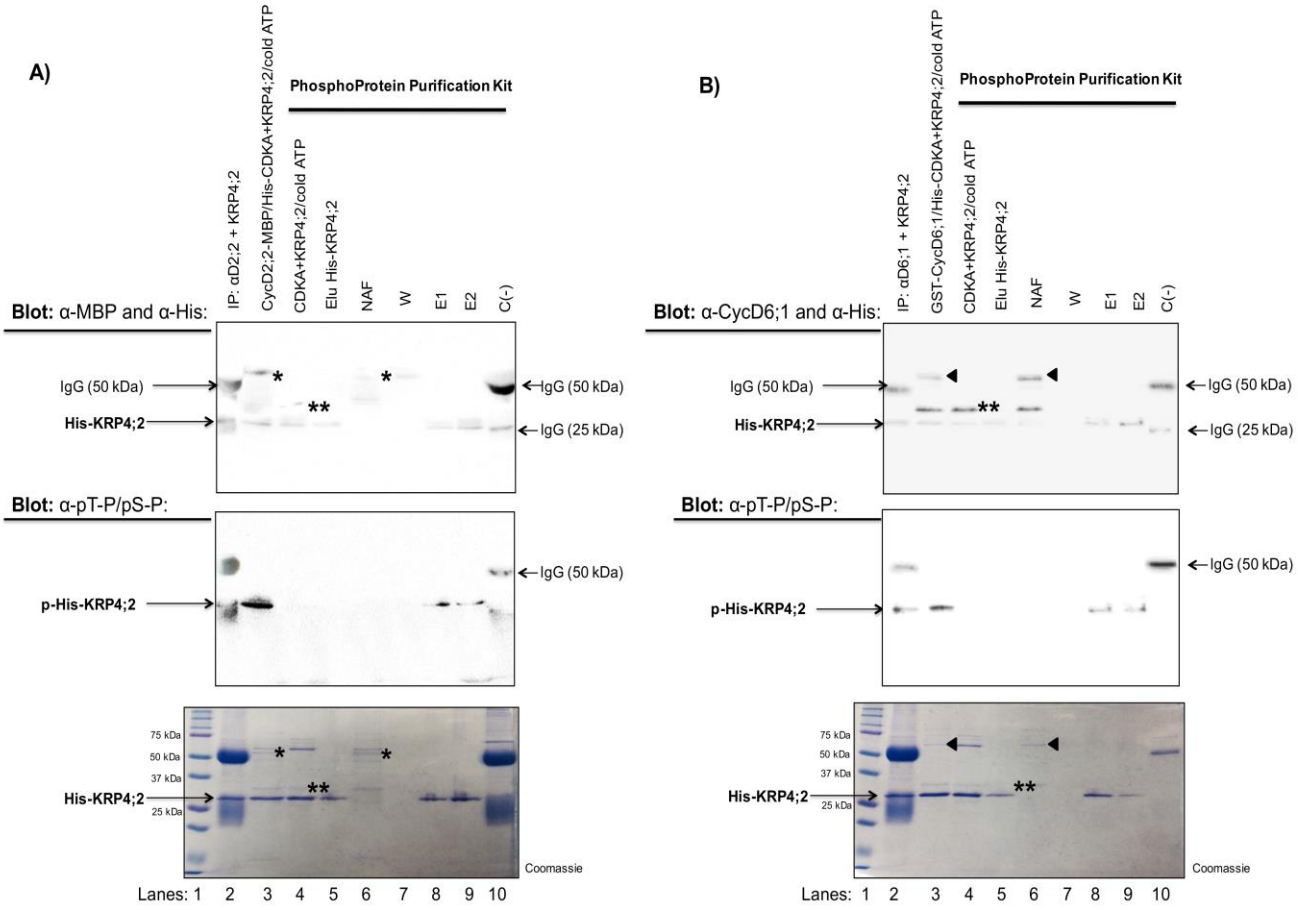
A)
PhosphoProtein Purification Kit
IP: αD2;2 + KRP4;2
CycD2;2-MBP/His-CDKA+KRP4;2/cold ATP
CDKA+KRP4;2/cold ATP
Elu His-KRP4;2
NAF
W
E1
E2
C(-)
Blot: α-MBP and α-His:
IgG (50 kDa)
His-KRP4;2
IgG (50 kDa)
IgG (25 kDa)
Blot: α-pT-P/pS-P:
p-His-KRP4;2
IgG (50 kDa)
75 kDa
50 kDa
37 kDa
25 kDa
His-KRP4;2
Coomassie
Lanes: 1 2 3 4 5 6 7 8 9 10
B)
PhosphoProtein Purification Kit
IP: αD6;1 + KRP4;2
GST-CycD6;1/His-CDKA+KRP4;2/cold ATP
CDKA+KRP4;2/cold ATP
Elu His-KRP4;2
NAF
W
E1
E2
C(-)
Blot: α-CycD6;1 and α-His:
IgG (50 kDa)
His-KRP4;2
IgG (50 kDa)
IgG (25 kDa)
Blot: α-pT-P/pS-P:
p-His-KRP4;2
IgG (50 kDa)
75 kDa
50 kDa
37 kDa
25 kDa
His-KRP4;2
Coomassie
Lanes: 1 2 3 4 5 6 7 8 9 10

Figure S8.

Supplementary Figure S8. Controls for purification of phosphorylated KRP4;2. **A)** Control for purification of KRP4;2 phosphorylated by CycD2;2-MBP/His-CDKA complex using PhosphoProtein Purification Kit. **B)** Control for purification of KRP4;2 phosphorylated by GST-CycD6;1/His-CDKA complex using PhosphoProtein Purification Kit. **Lane 1;** Protein molecular mass markers. **Lane 2;** Immunoprecipitation (IP) using anti-CycDs antibodies, His-KRP4;2 as substrate. **Lane 3;** His-KRP4;2 phosphorylation by the maize CycD/His-CDKA complex using cold ATP. **Lane 4;** Maize His-CDKA/His-KRP4;2 complex with cold ATP purified using the PhosphoProtein Purification Kit column. **Lane 5;** Elution of non-phosphorylated His-KRP4;2 from the PhosphoProtein resin, as negative control. **Lane 6;** Non-absorbed fraction (NAF) of CycDs-CDKA complexes with His-KRP4;2 after incubation with ATP to the PhosphoProtein resin. **Lane 7;** Washing steps. **Lanes 8-9;** Elutions 1 and 2 of p-His-KRP4;2. **Lane 10;** C(-) IP with anti-CycDs antibodies, no KRP4;2 added. Western Blots were performed using anti-MBP, anti-His, anti-CycD6;1 and anti pT-P/pS-P antibodies; CycD2;2-MBP is indicated with an asterisk, His-CDKA with two asterisks and CycD6;1 with an arrow head. Membranes stained with Coomasie Blue are added as loading control.

CycD2;2-MBP	-	+	+	+	-	-	-
GST-CycD6;1	-	-	-	-	+	+	+
His-CDKA	-	-	+	+	-	+	+
His-KRP4;2	+	+	+	-	+	+	-
p-His-KRP4;2 (CycD2;2-MBP/His-CDKA)	-	-	-	+	-	-	-
p-His-KRP4;2 (GST-CycD6;1/His-CDKA)	-	-	-	-	-	-	+

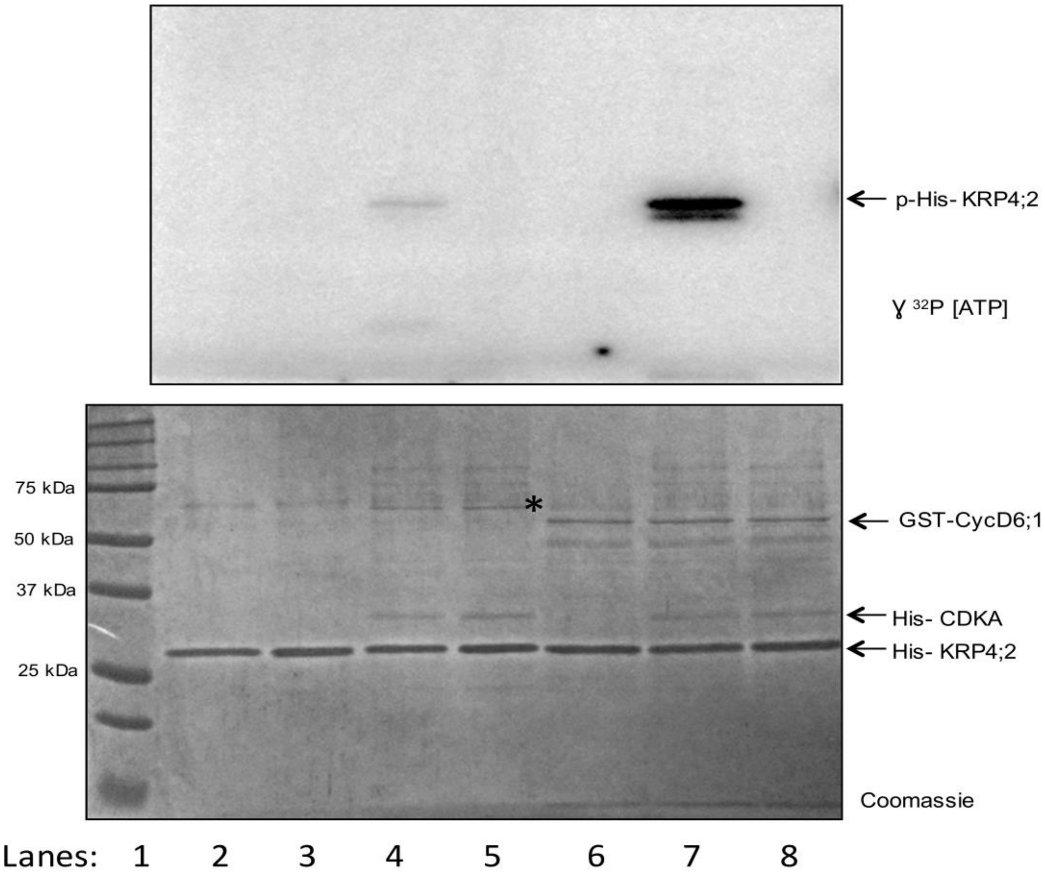

Figure S9.

Supplementary Figure. S9. No Phosphorylación of p-His-KRP4;2$^{\text{(CycD2;2-MBP/His-CDKA or GST-CycD6;1/His-CDKA)}}$ by recombinant CycD2;2-MBP-His-CDKA and GST-CycD6;1-His-CDKA complexes. **Lane 1;** Protein molecular mass markers. **Lane 2**; His-KRP4;2 in kinase buffer and radioactive ATP in the absence of a CycD-CDKA complex. **Lane 3**; His-KRP4;2 with CycD2;2-MBP in the absence of His-CDKA. **Lane 4**; His-KRP4;2 phosphorylation by CycD2;2-MBP-His-CDKA complex. **Lane 5;** p-His-KRP4;2 (phosphorylated by CycD2;2-His-CDKA, cold ATP) in the presence of CycD2;2-MBP-His-CDKA complex. **Lane 6;** His-KRP4;2 with GST-CycD6;1 in the absence of His-CDKA. **Lane 7**; His-KRP4;2 phosphorylation by GST-CycD6;1-His-CDKA complex. **Lane 8;** p-His-KRP4;2 (phosphorylated by GST-CycD6;1-His-CDKA, cold ATP) in the presence of GST-CycD6;1-His-CDKA complex. The Coomassie Blue stained gel was used as loading control. The band corresponding to CycD2;2-MBP is indicated with an asterisk. Representative image of three independent biological replicates.
